# Glutaminolysis impairment and immunometabolic dysregulation in U937 cells: Key mechanisms in occupational and environmental skin exposure to UV and benzo[a]pyrene

**DOI:** 10.1007/s00204-025-04155-4

**Published:** 2025-08-25

**Authors:** Christian Kersch, Viktor Masutin, Laura Kuhlmann, Rasha Alsaleh, Andrea Kaifie, Simone Schmitz-Spanke

**Affiliations:** Institute and Outpatient Clinic of Occupational, Social, and Environmental Medicine, Friedrich-Alexander-University of Erlangen-Nuremberg, Henkestr. 9–11, 91054 Erlangen, Germany

**Keywords:** Skin, Metabolomics, Immunometabolism, Glutaminolysis, Polycyclic aromatic hydrocarbons, UV, Lipid peroxidation, Monocytes, Ferroptosis, Lipids

## Abstract

Dermal exposure to polycyclic aromatic hydrocarbons (PAHs) and UV irradiation in occupational and environmental settings poses a health risk by inducing skin toxicity, including immunomodulatory effects. This study investigated the effects of benzo[a]pyrene (B[a]P), a well-characterized PAH, at three concentrations (0.04 nM, 4 nM, and 4 µM) and UV irradiation on human monocytic U937 cells, employing both single and combined exposure scenarios. An integrated metabolomics and toxicological approach was utilized to assess cellular responses, with a focus on understanding the immunometabolic effects of these exposures. Our findings revealed that only the highest B[a]P concentration in combination with UV irradiation resulted in significant metabolic dysregulation and impaired cellular function. Notably, we observed a pronounced downregulation of glutaminolysis, a critical metabolic pathway for cellular energy production and biosynthesis. This was evidenced by decreased levels of glutamate and key intermediates within the tricarboxylic acid cycle (e.g., succinate, fumarate, malate, and citrate), as well as reduced levels of glycine, a precursor for glutathione synthesis. In parallel, toxicological assays revealed increased levels of oxidative stress markers, lipid peroxidation, and enhanced DNA damage. Furthermore, the combined exposure led to alterations in tryptophan metabolism and dysregulation of lipid species, particularly sphingolipids and phosphatidylinositols. These findings lead us to propose the hypothesis that metabolic disruption, specifically the impairment of glutaminolysis, initiated a cascade of events, including increased oxidative stress, lipid peroxidation, and ultimately, ferroptosis in our study. Our results indicate that the combined exposure to UV irradiation and B[a]P can induce immunometabolic reprogramming and significantly contribute to the pathogenesis of inflammatory skin diseases.

**Supplementary Information:**

The online version contains supplementary material available at 10.1007/s00204-025-04155-4.

## Introduction

Polycyclic aromatic hydrocarbons (PAH) are occupational and environmental pollutants posing a significant threat to public health (Hartwig 2013). Both PAHs and UV irradiation are well-known inducers of oxidative stress, raising concerns about their combined effects on skin health beyond potential co-carcinogenic activity.

UV irradiation triggers reactive oxygen species (ROS) generation through a photochemical reaction with endogenous photosensitizers (Chen et al. 2021). Activation of benzo[a]pyrene (B[a]P), a well-studied PAH, can occur via metabolic or photochemical pathways. Metabolic activation by cytochrome P450 (CYP) enzymes generates reactive metabolites that interact with cellular macromolecules. Alternatively, photoexcited B[a]P directly interacts with molecular oxygen or biological molecules, leading to reactive intermediates (Fu et al. 2012; Penning et al. 1999).

While the toxic in particular DNA-damaging potential of individual and combined PAH and UV exposure is recognized, the dermatotoxic effects beyond cancer remain poorly understood. Both stressors possess immunomodulatory properties (Bernard et al. 2019). B[a]P, for instance, can disrupt the skin barrier and create an immunosuppressive tumor microenvironment through various mechanisms (Abd El-Fattah and Abdelhamid 2021; Malešević et al. 2024). B[a]P's role in monocytic cells is significant, with its toxic effects likely mediated by CYP and oxidative stress pathways (Ranjit et al. 2016). Through these mechanisms, PAHs are suspected of triggering or exacerbating inflammatory skin diseases (Jin et al. 2024). However, the precise mechanisms underlying these effects, especially with combined UV exposure as experienced by environmentally exposed individuals and outdoor workers, remain largely unknown.

While individual effects of PAHs and UV radiation on skin toxicity have been investigated, our understanding of their combined impact remains limited (Kersch et al. 2025; Weistenhofer et al. 2022). Moreover, metabolomic studies examining the individual and combined effects on skin, particularly within the context of immunometabolism, are scarce (Jiang et al. 2017; Masutin et al. 2024; Potratz et al. 2016; Randhawa et al. 2014; Yang et al. 2021).

In immune cells, the primary metabolic pathways are finely tuned to meet their specific needs. Alterations in these pathways can significantly influence immune cell function. For example, glycolysis provides rapid ATP and biosynthetic intermediates essential for quick responses, while the tricarboxylic acid (TCA) cycle supports efficient ATP production and cell longevity. The pentose phosphate pathway generates nucleotides and NADPH, aiding in cell proliferation and antioxidant defense. Fatty acid synthesis is vital for cell growth and inflammatory responses. Amino acid metabolism supports protein synthesis and feeds into other pathways, essential for immune cell activation and function (O'Neill et al. 2016; Palsson-McDermott and O'Neill 2025). Monocytes, for instance, undergo sequential metabolic and bioenergetic rewiring during acute inflammation, transitioning from anabolic activation to catabolic deactivation and early resolution (Zhu et al. 2019). To our knowledge, the effects of B[a]P and/or UV on these metabolic pathways have not yet been studied.

To investigate the effects on skin immune cells, for which the human monocyte cell line U937 serves as an in vitro model (e.g., as used in OECD Test No. 442E: In Vitro Skin Sensitization), we exposed these cells to a range of B[a]P concentrations, both individually and in combination with UV radiation. Our aim was to investigate how combined exposure compared to individual exposures affects monocyte function and whether a dose–response relationship could be observed. To achieve this, we combined an untargeted metabolomic and a targeted lipidomic approach with toxicological assays. By combining these approaches, we could gain a more complete picture of the cellular response to the exposure, linking changes in cellular metabolism to observed effects on cell function.

## Materials and methods

### Chemicals and reagents

The following reagents were used for cell culture, exposure, and sample preparation: standard chemicals for cell culture c.c.pro GmbH (Gesellschaft für Herstellung und Vertrieb von Produkten für Cellculturen mbH, Am Bahnhof 1, 99,986 Oberdorla, Germany); B[a]P Merck KGaA, (Darmstadt, Germany, CAS: 50–32-8); and dimethyl sulfoxide (DMSO) AppliChem GmbH (Darmstadt, Germany, CAS: 67–68-5). Other chemicals were obtained from Alfa Aesar by Thermo Fisher (Kandel) GmbH (Kandel, Germany); Merck KGaA (Darmstadt, Germany); Life Technologies by Thermo Fisher Scientific (Waltham, USA); Promega GmbH (Walldorf, Germany); Santa Cruz Biotechnology, Inc. (Heidelberg, Germany); and Carl Roth GmbH + Co. KG (Karlsruhe, Germany). All chemicals were used at the highest available level of purity.

### Cell culture and exposure

U937 cells were cultured in RPMI 1640 medium supplemented with 10% fetal calf serum, 1% L-glutamine, and 1% penicillin/streptomycin. After seeding at a density of 1 × 10^6^ cells per well in 96-well and 6-well plates, cultures were incubated at 37 °C in a humidified atmosphere containing 5% CO_2_. A 24-h adhesion period preceded experimental treatments. Given the limited penetration depth of UVB into tissues and the observed sensitivity of U937 cells to UVA in preliminary experiments, cells were exposed to a single dose of 1.7 J/cm^2^ UVA using a BS-02 UV/VIS UV LED chamber (see Supplementary Information for details). This chamber emits wavelengths between 280 and 400 nm, and light dosage was precisely monitored with integrated RM-12 radiometric sensors. To investigate dose–response relationships, cells were treated with 0.04 nM, 4 nM, or 4 µM B[a]P. For combined exposure experiments, cells were immediately irradiated after exposure to B[a]P.

### Metabolomics

Three biological replicates were used for all metabolomic analyses (GC–MS, targeted lipidomics).

#### Untargeted metabolomics (GC–MS)

##### Sample preparation

Following exposure, cells were washed and immediately stored. Metabolite extraction was performed using a biphasic method with cold methanol and methyl tert-butyl ether (MTBE), followed by homogenization and phase separation. Both aqueous and organic fractions were collected, dried, and subsequently derivatized to prepare them for analysis. Finally, the derivatized aqueous and organic fractions were combined and analyzed by gas chromatography-mass spectrometry (GC–MS). Detailed information is provided in the Supporting Methods.

##### GC–MS analysis

Derivatized metabolites were analyzed using an Agilent 8890 Gas Chromatograph (GC) system coupled to an Agilent 5977B quadrupole mass spectrometer (GC–MS). Chromatographic separation was achieved on an OPTIMA 5 MS column (30 m × 0.32 mm inner diameter, 0.25 μm film thickness), a nonpolar-low polar column. Two microliters of each sample was injected in splitless mode into the GC inlet, maintained at 250 °C with a total flow rate of 54.5 mL/min. Helium was used as the carrier gas at a constant flow rate of 1.5 mL/min in isobaric mode. The GC oven temperature was initially held at 80 °C for 10 min, then ramped at a rate of 5°C/min to 330 °C, and finally held at 330 °C for 10 min.

##### Data processing and analysis

Raw mass spectrometry (GC–MS) data underwent peak deconvolution and identification of metabolic signatures using R Studio with the annotated Golm Metabolome Database (GMD). Data were then normalized, log10 transformed, and Pareto scaled. All statistical analyses, including univariate comparisons (e.g., t-tests or ANOVA) for individual differentially regulated metabolites, multivariate analysis (principal component analysis (PCA), orthogonal partial least squares discriminant analysis (OPLS-DA), variable importance in projection (VIP)), as well as pathway analysis, were performed using MetaboAnalyst 6.0. Data visualization utilized SankeyMATIC software (for detailed information see Supporting Methods).

#### Targeted metabolomics (lipidomics)

##### Sample preparation

Following exposure, cells were promptly quenched and underwent a standardized lysis procedure involving repeated freeze–thaw cycles, centrifugation, and sonication. Metabolites were then extracted using a methanol-based protocol to precipitate proteins and isolate polar compounds. The extracts were dried under vacuum, resuspended in a multi-solvent system, and prepared for subsequent analysis. For targeted lipidomics, lipids of particular interest were identified and selected based on a thorough literature review (further information are provided in the Supporting Methods).

##### LC–MS/MS analysis

Liquid chromatography-mass spectrometry (LC–MS) analysis was performed using a Waters ACQUITY UPLC H-Class system coupled to a Waters Xevo TQ-XS triple quadrupole mass spectrometer. Chromatographic separation was achieved on an ACQUITY HSS T3 column with a gradient elution program. The mass spectrometer was operated in multiple reaction monitoring (MRM) mode with alternating positive and negative electrospray ionization. Detailed instrumental parameters for LC-MS analysis, including mobile phase compositions and gradient program, are provided in Supporting Methods.

### Toxicological assays

The toxicological impact of exposures was comprehensively evaluated through a series of established assays. Oxidative stress was assessed by measuring reactive oxygen species (ROS) production, glutathione redox status (GSH/GSSG ratio), lipid peroxidation (malondialdehyde formation), and the activity of the phase II antioxidant enzyme NQO1. Cell viability was determined via lactate dehydrogenase (LDH) release, indicative of membrane damage, while mitochondrial function was evaluated by assessing metabolic activity (MTT reduction) and mitochondrial membrane potential (MMP). DNA damage was quantified using the alkaline comet assay (detailed information are provided in the Supporting Methods).

### Statistical analysis

Statistical significance was determined by ANOVA with post hoc Scheffé test (*p* < 0.005). Data are presented as mean ± SEM (*n* = 4 technical, *n* = 4 biological replicates; 3 technical replicates were performed for the COMET analyses).

## Results

### Metabolic profiling reveals significant metabolite alterations only upon combined exposure

To obtain a comprehensive overview of the metabolic changes resulting from the different exposures, we first conducted untargeted metabolomics. This allowed us to identify significantly regulated metabolites within each experimental group, which were then compared across all conditions. A total of 980 unique metabolite hits were identified in U937 cells, with 85 metabolites assigned a match factor (MF) greater than 0.9 (Supplementary Information Table S1). Volcano plot analysis identified three metabolites significantly upregulated following UV irradiation compared to control condition: glucose-1-phosphate, L-glutamic acid, and mandelic acid (Table S2). Exposure with 4 µM B[a]P regulated six metabolites, with two downregulated (indoleacetaldehyde and 5-hydroxy-L-tryptophan) and four upregulated (palmitic acid, fumarate, 3-sulfonalanine, and glutamate) compared to the control (Table S3). Combined 4 µM B[a]P and UV exposure elicited a more pronounced metabolic response, with 41 metabolites downregulated and 5 upregulated compared to control condition. Among them, 5-methylthioadenosine, glutaric acid, fumarate, and glutamine were strongly downregulated, while ƴ-linolenic acid, galactose, and mandelic acid were significantly upregulated. Interestingly, glutamate was downregulated in this group, contrasting its upregulation in the other exposure conditions (Tables S4).

We employed principal component analysis (PCA), to discriminate the regulated metabolites of the single (4 µM B[a]P) and combined exposure groups from those of the control conditions. The PCA plot clearly differentiated B[a]P and UV-exposed samples from the single exposed and control groups, suggesting a pronounced effect of the combined exposure on the metabolite profiles. Additionally, the plot indicated that individual exposures to UV or B[a]P did not significantly alter the metabolic profile in our experimental setting (Supplementary Information Figure S1). To identify common and unique regulated metabolites across the groups, orthogonal partial least squares discriminant analysis (OPLS-DA) was conducted, followed by the calculation of variable importance in projection (VIP) scores to assess metabolite contributions to the group separation (Figure S2).

As a complementary analysis to the volcano plot and VIP score, a Venn diagram was employed to provide a comprehensive overview of the metabolic changes across all experimental groups (Figure S3). Most notably, 40 metabolites were significantly differentially regulated (*p* < 0.1) exclusively in the combined exposure group, with no overlap observed with the other experimental groups. The combined exposure downregulated 37 metabolites, while 3 metabolites (docosahexaenoic acid, γ-linolenic acid, and galactose) were upregulated. This implies a distinct regulatory response in the combined exposure group, which is consistent with the PCA analysis. The B[a]P group uniquely featured 4 metabolites (5-hydroxy-L-trytophan, indoleacetaldehyde, palmitic amide, 3-sulfinoalanine). Mandelic acid and glucose-1-phosphate were shared between the UV and the combined exposure groups, while fumarate was unique to the B[a]P and the combined exposure groups. Notably, glutamate was common to all groups but exhibited a significant downregulation after combined exposure in contrast to an upregulation after single UV and B[a]P exposure (Figure S3).

### Glutamine-centered metabolism under B[a]P and UV

To further investigate the biological implications of the differentially regulated metabolites, we mapped them to known metabolic pathways. By constructing a Sankey diagram, we visualized the flow of regulated metabolites through these pathways, providing insights into the underlying biological processes that may be regulated (Figure S4). This diagram revealed distinct metabolic profiles for each exposure group. Notably, the combined exposure to B[a]P and UV resulted in a marked enrichment of metabolites involved in alanine, aspartate, and glutamate metabolism, suggesting a central role for glutamine in the metabolic response.

**Fig. 1:**
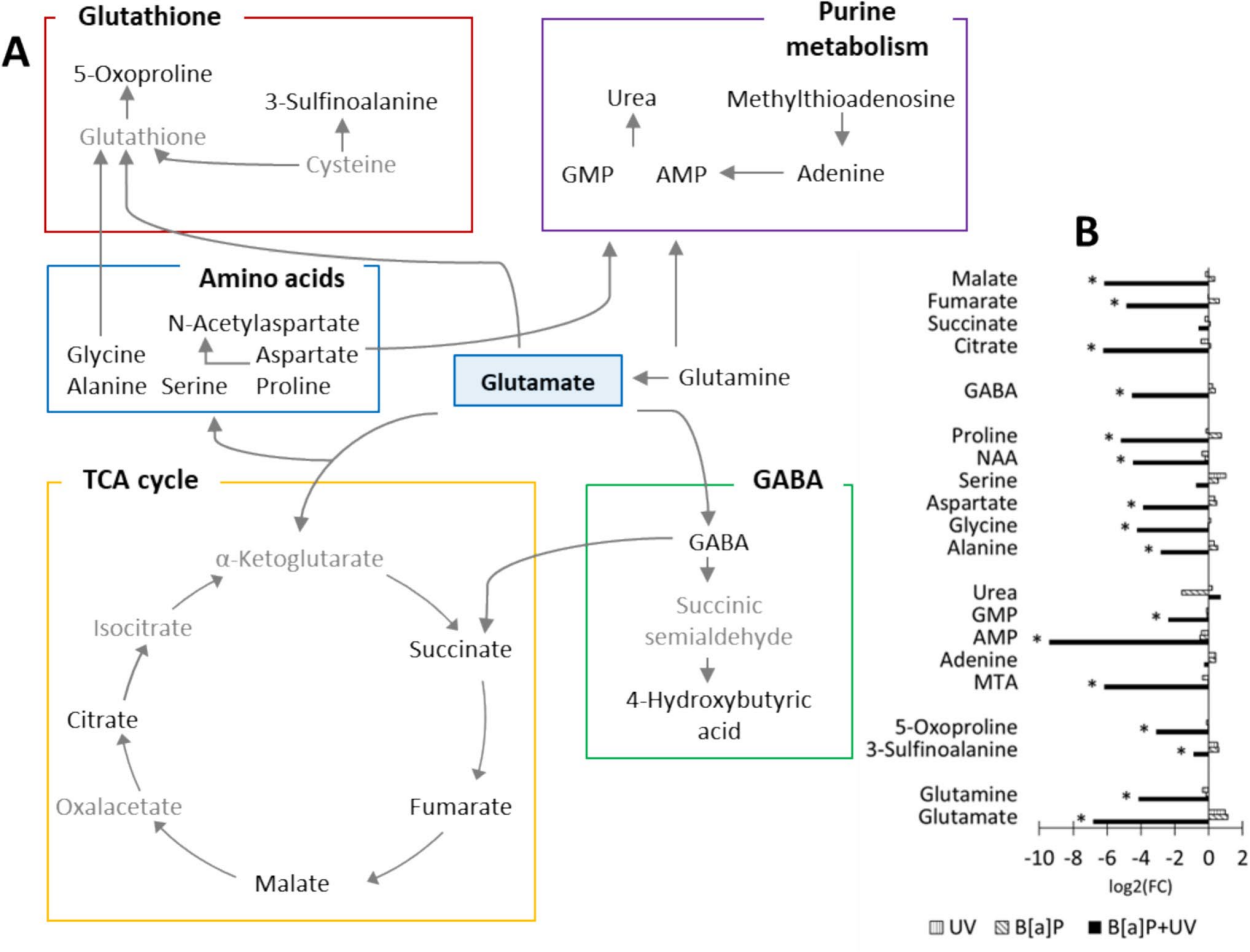
**A** a schematic overview of the pathways influenced by glutamate in our study. Detected metabolites are highlighted in black, while undetected metabolites are shown in gray. **B** the log2 fold change of significantly altered metabolites (*p* < 0.05) between the treatment and control. Statistical significance was determined using one-way ANOVA followed by post hoc Fisher's LSD test. *GABA* γ-aminobutyric acid, *GMP* guanosine monophosphate, *AMP* adenosine monophosphate, *NAA* N-acetyl-L-aspartic acid, *MTA* methylthioadenosine, *5-oxoproline *pyroglutamic acid

Glutamine is a central regulator of cellular amino acid pools and redox balance. Figure 1A illustrates the regulated pathways (glutathione metabolism, purine metabolism, amino acid biosynthesis, TCA cycle, and γ-aminobutyric acid (GABA)) in the exposed groups, highlighting significant metabolic shifts compared to control. Glutamine serves as a precursor for α-ketoglutarate, a key TCA cycle intermediate, and is converted to various amino acids via transamination. Glutathione, a major antioxidant, is synthesized from glycine, glutamate, and cysteine. Aspartate and glutamate contribute to nucleotide biosynthesis. Additionally, glutamate is the precursor of GABA, a neurotransmitter implicated in diverse physiological processes, including immune regulation, glucose homeostasis, and inflammation (Huang et al. 2019).

The importance of glutamine-dependent pathways in response to the combined exposure is highlighted by the numerous significantly regulated metabolites within these pathways. Notably, the combined exposure significantly downregulated a majority of metabolites (Fig. 1B).

### Altered tryptophan metabolism in response to combined exposure

**Fig. 2:**
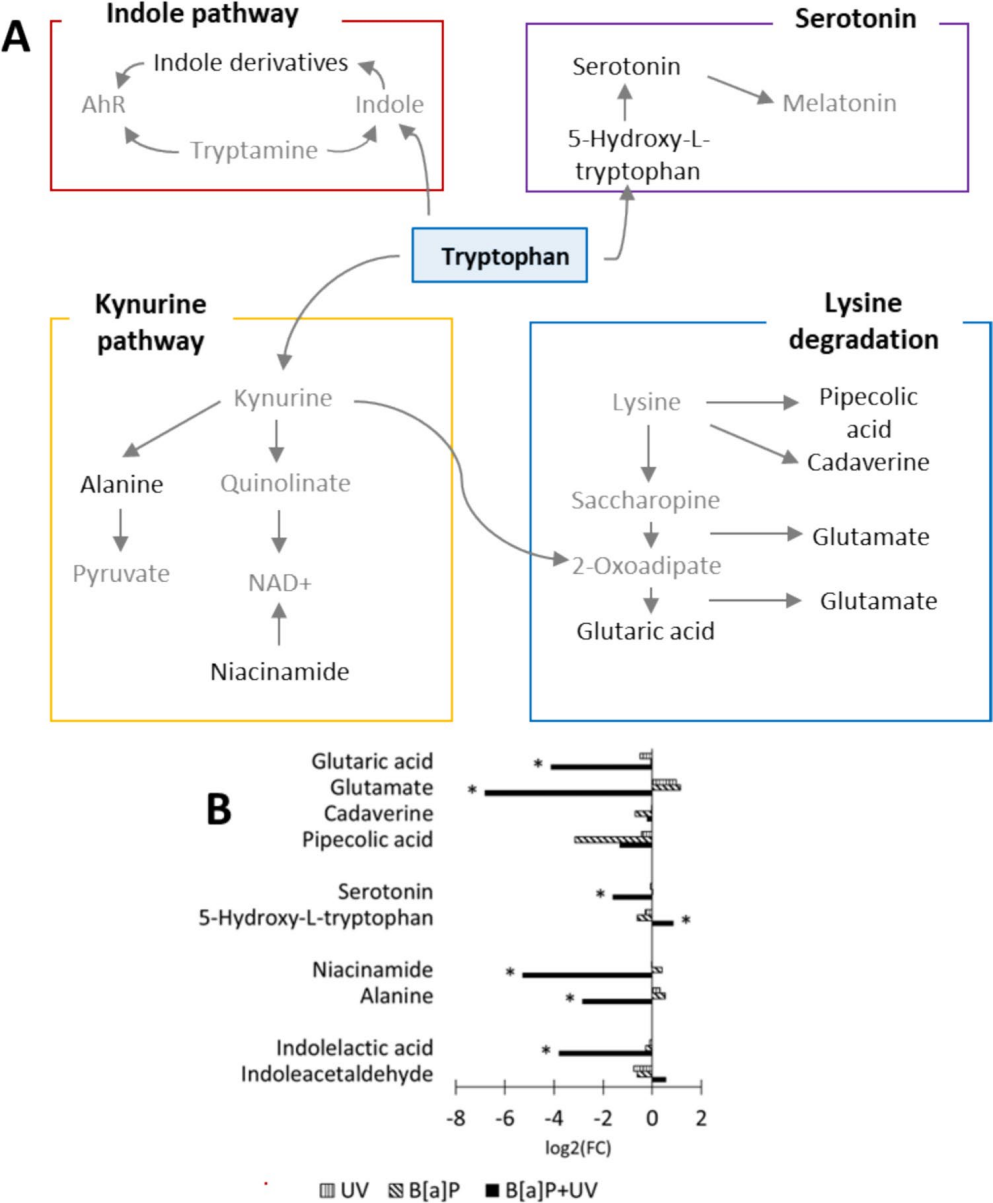
**A** a schematic overview of the pathways influenced by tryptophan in our study. Detected metabolites are highlighted in black, while undetected metabolites are shown in gray. **B** the log2 fold change of significantly altered metabolites (*p* < 0.05) between the treatment and control. Statistical significance was determined using one-way ANOVA followed by post hoc Fisher's LSD test. *AhR* aryl hydrocarbon receptor, *NAD + * nicotinamide adenine dinucleotide

The tryptophan metabolism generates diverse bioactive compounds that influence a range of physiological processes, including inflammation, metabolism, immune responses, and neurological function (Xue 2023). Our Sankey and Venn diagram analyses revealed significant regulatory alterations within the tryptophan pathway (Figure S3 and S4). Specifically, we observed downregulation of indolelactic acid, a ligand for the aryl hydrocarbon receptor (AhR), in the indole pathway following combined exposure (Xue et al. 2023). In the serotonin pathway, both 5-hydroxy-L-tryptophan and serotonin were significantly regulated. Notably, only 5-hydroxy-L-tryptophan was upregulated following the combined exposure. While no direct tryptophan metabolites were detected in the kynurenine pathway, nicotinamide (niacinamide) and quinolinate, precursors of NAD^+^, were downregulated. As NAD^+^ is a cofactor in redox reactions, DNA repair, and cell signaling, its reduction may have broad implications. Additionally, the downregulation of metabolites in the kynurenine pathway, associated with lysine degradation and glutamate metabolism, highlights the interconnectedness of these pathways (Fig. 2A and B).

### Untargeted and targeted lipidomics reveal complex lipid responses to combined exposure

We combined untargeted GC–MS with a targeted LC–MS/MS approach to comprehensively analyze lipid (components) changes.

**Fig. 3:**
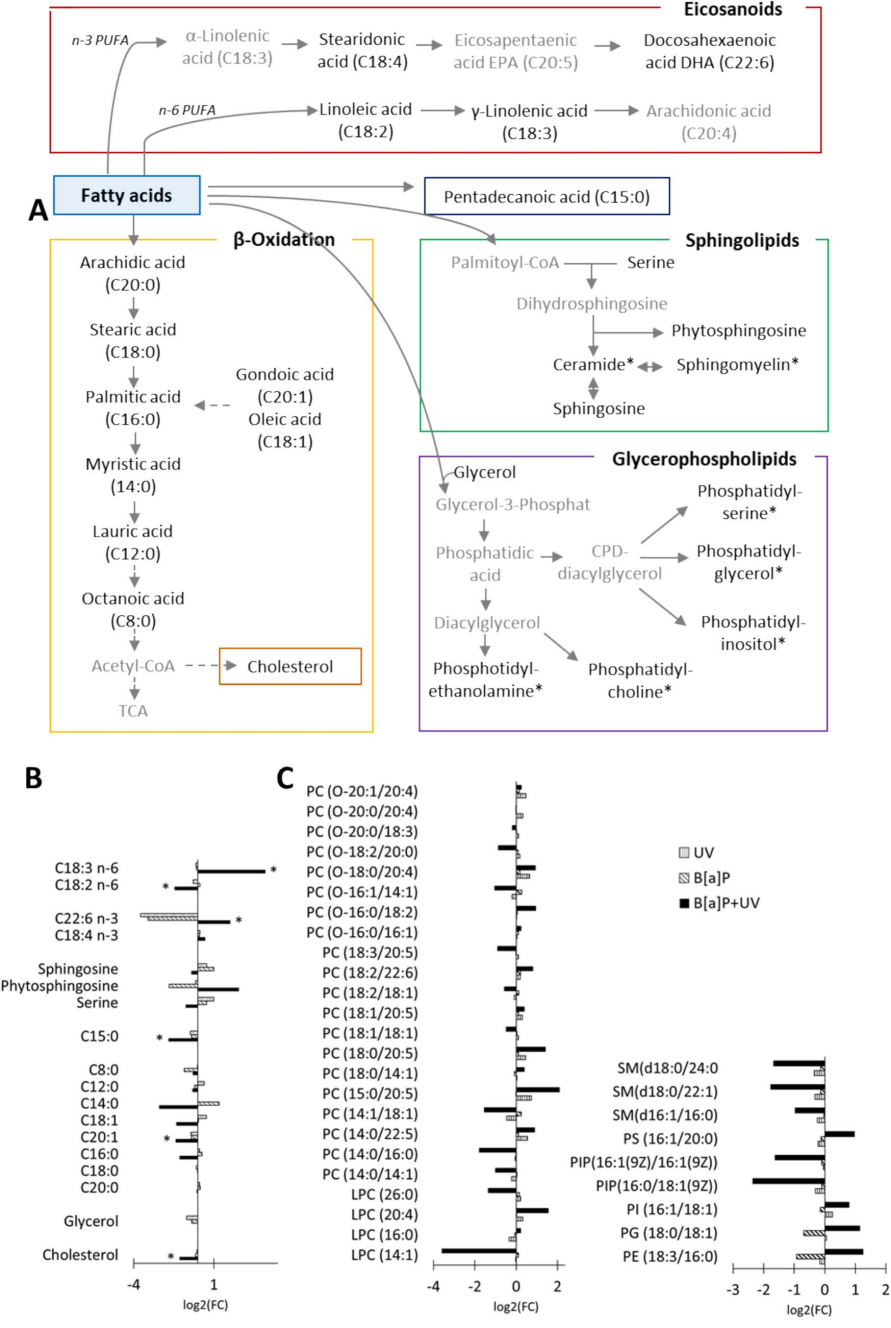
**A** a schematic overview of the altered lipid pathways in our study. Detected lipids are highlighted in black, while undetected metabolites are shown in gray. The lipids marked with * were quantified using the targeted approach. **B** the log2 fold change of significantly altered lipids of the untargeted approach (*p* < 0.05) between the treatment and control. Statistical significance was determined using one-way ANOVA followed by post hoc Fisher's LSD test. **C** the log2 fold change of the significantly regulated lipids of the targeted approach. Statistical significance was determined using one-way ANOVA followed by post hoc Fisher's LSD test. *TAG* triacylglycerol, *TCA* tricarboxylic acid cycle, *PUFA* polyunsaturated fatty acid

While the untargeted analysis revealed only a few significantly regulated metabolites, the contrasting effects of the different exposures on docosahexaenoic acid (DHA) and the arachidonic acid precursors were notable (Fig. 3 A and B). DHA displayed a distinct pattern, being upregulated after combined exposure but downregulated after single exposures. Linoleic acid was downregulated, and γ-linolenic acid was upregulated following the combined exposure. These eicosanoid fatty acids, belonging to the polyunsaturated fatty acid (PUFA) family, function as lipid mediators, exhibiting both pro- and anti-inflammatory properties (Tyurina et al. 2019). The observed changes in the regulation of fatty acids involved in β-oxidation indicate a decreased rate of β-oxidation following combined exposure (Fig. 3B).

The targeted analysis was performed to quantify sterols, glycerolipids, sphingolipids, and specific subclasses of glycerophospholipids (Fig. 3). Table S11 presents the lipids quantified using this approach, while Table S12 displays the significantly regulated ones. Based on volcano plot analysis, Tables S13–S15 provide a list of lipids that exhibited significant differential regulation in each exposure group relative to the control. Consistent with the previous analyses, targeted lipidomic profiling demonstrated a distinct clustering of lipid profiles in the combined exposure group as revealed by PCA (Figure S8). Venn diagram analysis indicated minimal overlap between all groups, and volcano plot analysis identified only a limited number of significantly regulated lipids in the individual exposure groups. The combined exposure group exhibited the most pronounced changes in lipid abundance, with LPC (14:1) showing the greatest decrease and LacCer (d18:1/22:1) the most significant increase (Figure S9). The hypothesis of increased LacCer degradation is supported by the significantly elevated galactose levels (see chapter on metabolites not assigned to a pathway).

The magnitude of lipid regulation was generally lower compared to that observed for metabolites in the untargeted analysis. ANOVA followed by post hoc Fisher's LSD test revealed significant lipid regulations as depicted in Fig. 3C. While no consistent trend was observed across individual lipid (sub)classes, sphingomyelins (SM) and phosphatidylinositol phosphates (PIP) exhibited a consistent downregulation following combined exposure. The reduced concentrations of inositol and inositol-2-phosphate, precursors of phosphatidylinositol phosphates, suggest a decreased production of these lipids (as shown in the chapter on unassigned metabolites).

### Metabolites not assigned to specific pathways

Figure S5 highlights metabolites of the untargeted metabolic profiling that exhibited significant regulation but did not align with any of the previously described pathways. The identified metabolites encompass a variety of amino acids (valine, norleucine, threonine, asparagine, isoleucine, alanylalanine), urea cycle intermediates (ornithine, homocitrulline), and compounds involved in glycolysis (galactose, lactate), the inositol pathway (inositol, inositol 2-phosphate), and DNA/RNA metabolism (thymine).

### Combined exposure induces cytotoxicity, oxidative stress, and DNA damage: measurement of toxicological endpoints

**Fig. 4:**
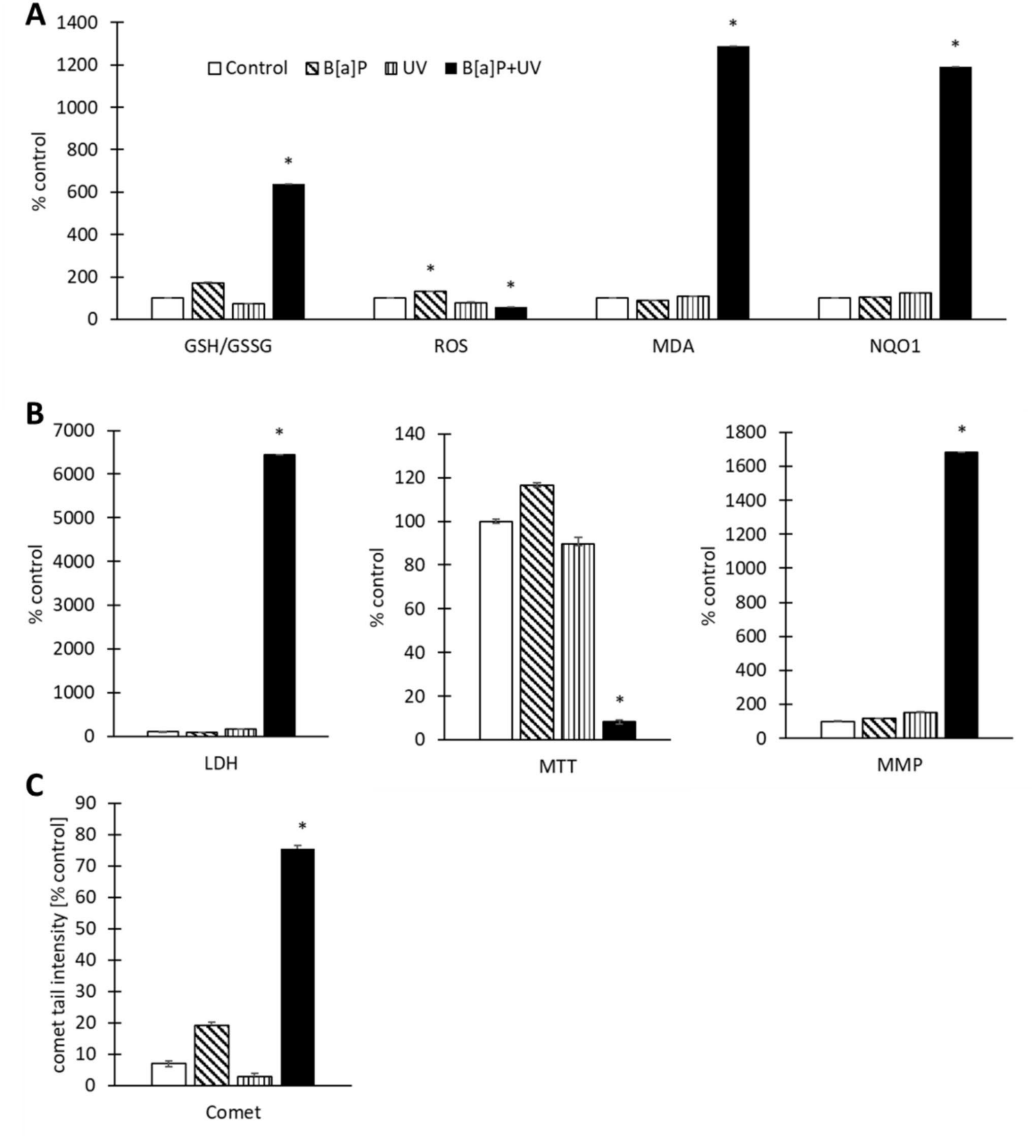
Effects of exposures on **A** oxidative status in terms of GSH/GSSG ratio, ROS, MDA, NQO1, **B** viability/cytotoxicity in terms of LDH, MTT, MMP, and (**C**) DNA damage, normalized to the control condition. Data are presented as mean ± SEM (*n* = 4 technical, *n* = 4 biological replicates; 3 technical replicates were performed for the COMET analyses). Statistical significance was determined by ANOVA with post hoc Scheffé test (*p* < 0.005). GSH/GSSG, reduced/oxidized glutathione; *ROS* reactive oxygen species, *MDA* malondialdehyde; *NQO1* NAD(P)H:quinone oxidoreductase 1, *LDH* lactate dehydrogenase, *MTT* 3-(4,5-dimethylthiazol-2-yl)−2,5-diphenyltetrazolium bromide, *MMP* mitochondrial membrane potential

In order to link the metabolomics data with potential toxicological effects, we performed a series of assays (Fig. 4, Table S17). Combined exposure to 4 µM B[a]P and UV significantly exacerbated oxidative stress, as indicated by altered GSH/GSSG ratio, elevated MDA levels, and decreased NQO1 activity. Intriguingly, ROS levels do not align with the other paramters of oxidative stress. Following combined exposure, ROS levels are paradoxically reduced compared to controls. While increased cytotoxicity might offer a partial explanation, the substantial elevation of all other parameters strongly suggests a pronounced state of oxidative stress.

Cellular viability and cytotoxicity, assessed by LDH release, MTT reduction, and MMP, were significantly compromised in the B[a]P + UV group. Furthermore, DNA damage, as measured by comet assay, was significantly augmented in the B[a]P + UV group compared to control.

These findings suggest that the combined exposure to B[a]P and UV irradiation induces a synergistic effect, leading to pronounced oxidative stress, cytotoxicity, and DNA damage in the cells.

### Dose–response relationship of combined B[a]P and UV exposure on cellular metabolism

Lastly, we aimed to elucidate the dose–response relationship of combined B[a]P and UV exposure at the metabolic level, particularly focusing on whether significant effects could be observed at lower B[a]P concentrations. To this end, we exposed cells to a range of B[a]P concentrations (0.04 nM, 4 nM, and 4 µM) in combination with UV irradiation. Notably, 25 metabolites were differentially regulated in a dose-dependent manner, with the most pronounced effects observed at the highest B[a]P concentration (4 µM) (Table S8). Principal component analysis and volcano plot analysis further highlighted the distinct metabolic profiles induced by the highest combined exposure (Tables S9–10, Figure S6, exposure to medium B[a]P combined with UV did not result in significant regulation). The Venn diagram demonstrates a clear dose-dependent effect of combined B[a]P and UV exposure on metabolite regulation. While low and medium B[a]P concentrations had minimal impact, the high B[a]P concentration significantly altered metabolite profiles, with AMP and citrate being most strongly downregulated and γ-linolenic acid and galactose being most strongly upregulated (Figure S7).

## Discussion

A limited number of metabolomic studies have been conducted to assess the individual, and fewer yet the combined, effects of UV radiation and polycyclic aromatic hydrocarbons on skin biopsies, keratinocytes, or fibroblasts (Jiang et al. 2017; Masutin et al. 2022, 2024; Potratz et al. 2016; Randhawa et al. 2013, 2014; Yang et al. 2021). While previous studies have provided valuable insights into the metabolic responses of skin cells to UV radiation and/or PAHs, no studies have yet investigated the impact of these stressors on the skin immune system, particularly in combination with toxicological endpoints.

Integrated metabolomic and endpoint analyses revealed that significant dysregulation in U937 cells occurred exclusively in the treatment group exposed to the highest B[a]P concentration in combination with UV irradiation. This observation suggests that U937 cells exhibit a degree of resilience to the investigated stressors under specific exposure conditions. Notably, neither B[a]P exposure at this concentration alone nor combined exposures with lower B[a]P concentrations induced significant cellular dysregulation.

Therefore, the question arises as to which pathways enable the cell to maintain its functionality under these conditions and which mechanisms underlie the observed adverse effects. Our findings from various analyses converge on glutamine as a pivotal molecule in the cellular response. Single exposure to B[a]P increased glutamate levels, whereas combined exposure decreased them. Glutaminolysis is a mitochondrial metabolic pathway that generates cellular energy through the degradation of glutamine (Mirveis et al. 2023). Glutamine is initially metabolized to glutamate, which undergoes oxidative deamination to form α-ketoglutarate. This key intermediate of the TCA cycle fuels oxidative phosphorylation, leading to ATP production. In our study, we observed significant regulation of TCA cycle metabolites, including succinate, fumarate, malate, and citrate, accompanied by a substantial decrease in MTT in the cytotoxicity assay. MTT reduction correlates with cellular metabolic activity, specifically with the levels of NADH and NADPH. NADH is primarily produced in the TCA cycle. The observed decrease in MTT suggests a reduction in mitochondrial function, which could be attributed to alterations within the TCA cycle. Beyond energy production, TCA cycle intermediates, such as fumarate, play a significant role in regulating immune response (Palsson-McDermott and O'Neill 2025). Our findings, which reveal impaired glutaminolysis, including fumarate metabolism, and decreased cholesterol levels, strongly suggest that these metabolic pathways are critical for monocyte function (Arts et al. 2016). Consequently, the combined exposure disrupts essential immune pathways. Given the involvement of fumarate in diseases like systemic lupus erythematosus, systemic sclerosis, and psoriasis, we hypothesize that this combined exposure could also have implications for these disorders (Palsson-McDermott and O'Neill 2025; Steadman and O'Reilly 2025).

Glutamine is also a precursor for glutathione, an important antioxidant. The significant downregulation of glycine and 5-oxoproline, indicative of potential glutathione depletion, combined with the increased GSH/GSSG ratio, MDA, and NQO1 levels, supports the hypothesis of increased oxidative stress. These findings suggest that the antioxidant defense system, particularly glutathione-dependent pathways, may be also compromised under these conditions.

In addition, glutaminolysis supports anabolic processes by providing carbon skeletons for amino acid synthesis. These include glycine, alanine, proline, and aspartate in our study, which are essential for nucleotide biosynthesis (Altman et al. 2016; Mirveis et al. 2023). Consistent with this, we observed significant regulation of GMP and AMP. Moreover, the combined exposure resulted in increased DNA damage, which may not have been efficiently repaired due to the impaired glutaminolysis.

Impaired glutaminolysis, as previously described, led to elevated MDA levels, indicative of increased lipid peroxidation. This heightened oxidative stress likely contributes to ferroptosis. PUFAs, with their multiple double bonds, are highly susceptible to oxidative attack by ROS. Consistent with this, we observed significant alterations in the levels of arachidonic acid precursors, linoleic acid (C18:2) and γ-linolenic acid (C18:3), as well as DHA following combined exposure, suggesting a potential contribution to inflammatory responses (Ziboh et al. 2000). Pronounced alterations were evident in sphingomyelins and ceramides. Ceramide can be generated through de novo synthesis, sphingosine salvage, or sphingomyelin hydrolysis. Previous studies have demonstrated an association between increased ceramide content and reduced sphingomyelin levels often observed in the context of oxidative stress and lipid peroxidation (Supruniuk et al. 2023). This relationship was also observed in our study.

Furthermore, several phospholipids significantly regulated in our study, including PC (18:2/22:6), PC (18:0/20:5), and PC (15:0/20:5), have been previously identified as drivers of ferroptosis, a nonapoptotic programmed cell death pathway (Qiu et al. 2024). These findings collectively suggest the hypothesis that the observed impairment of glutaminolysis initiates a cascade of events, culminating in increased lipid peroxidation and the induction of ferroptosis.

While ferroptosis has emerged as a player in skin diseases, investigations into ferroptosis induced by stressors beyond UV irradiation in the skin remain scarce (Wang et al. 2024). For instance, keratinocyte death via ferroptosis is described to initiate skin inflammation following UVB exposure (Vats et al. 2021). Moreover, the role of ferroptosis in myeloid cells, such as macrophages and monocytes, has been less extensively explored across various organ systems (Tang et al. 2021). For instance, in microglia, one study hypothesized that fatty acid oxidation modulates the susceptibility of activated M1 macrophages to undergo ferroptosis (Kapralov et al. 2020).

Beyond the regulation of glutaminolysis, our data highlight a significant impact of the combined exposure on tryptophan metabolism. Tryptophan metabolism governs key physiological processes, including inflammation and immunity (Xue et al. 2023). Notably, our study observed a significant downregulation of indole derivatives following combined exposure. The indole pathway, known for its critical role in intestinal function, is also active in macrophages and other cell types (Shatova and Shestopalov 2023; Sukka et al. 2024). Indole derivatives, acting in part as ligands for the AhR (as well as B[a]P), modulate immune responses (Jia et al. 2024). The AhR, in turn, regulates the expression of phase I enzymes, which are crucial for the detoxification of PAHs, while also exerting a modulatory influence on the immune system (Jia et al. 2024; Xue et al. 2023). Our findings may indicate that the combined exposure compromises the immune function of monocytes, likely due to the depletion of indole derivatives.

Furthermore, we should briefly discuss the downregulation of phosphatidylinositol phosphates (PIPs) after combined exposure. PIPs play a significant role in various cellular processes, particularly cell motility and adhesion, both of which are central to monocyte function. A further point is the downregulation of PIPs after combined exposure. PIPs are involved in numerous cellular processes, particularly cell motility and adhesion – two crucial aspects for monocyte function (Hou et al. 2025). Overall, the observed lipid regulations will significantly influence this aspect.

This study provides novel insights into the metabolic and toxicological consequences of combined exposure to UV irradiation and B[a]P on the human monocytic cell line U937. Our integrated metabolomics and toxicological analysis demonstrates that glutaminolysis and tryptophan metabolism are crucial for monocyte function. Impaired glutaminolysis, likely leading to increased lipid peroxidation and ferroptosis, may contribute to the pathogenesis of inflammatory skin diseases. However, it is important to note that further direct investigations to unequivocally confirm ferroptosis were not performed in this study. The interpretation of our findings should also consider that this study utilized the U937 cell line as an in vitro model to investigate the impact on skin immune cells. While U937 cells are a useful tool for studying monocytic responses, they do not fully represent the complexity of the skin's immune system within its native tissue environment. Nevertheless, these findings highlight the potential for combined occupational and environmental stressors to induce immune dysfunction and emphasize the need for further investigation into the mechanisms underlying these effects.

## Supplementary Information

Below is the link to the electronic supplementary material.

Supplementary file1 (DOCX 1456 KB)

## Data Availability

The authors confirm that the data supporting the findings of this study are available in the supplementary materials. Additional Inforamtion can be obtained from the corresponding author, upon reasonable request.

## References

[CR1] Abd El-Fattah EE, Abdelhamid AM (2021) Benzo[a]pyrene immunogenetics and immune archetype reprogramming of lung. Toxicology 463:152994. 10.1016/j.tox.2021.152994 34678320 10.1016/j.tox.2021.152994

[CR2] Altman BJ, Stine ZE, Dang CV (2016) From Krebs to clinic: glutamine metabolism to cancer therapy. Nat Rev Cancer 16(10):619–634. 10.1038/nrc.2016.71 27492215 10.1038/nrc.2016.71 PMC5484415

[CR3] Arts RJ, Novakovic B, Ter Horst R et al (2016) Glutaminolysis and fumarate accumulation integrate immunometabolic and epigenetic programs in trained immunity. Cell Metab 24(6):807–819. 10.1016/j.cmet.2016.10.008 27866838 10.1016/j.cmet.2016.10.008 PMC5742541

[CR4] Bernard JJ, Gallo RL, Krutmann J (2019) Photoimmunology: how ultraviolet radiation affects the immune system. Nat Rev Immunol 19(11):688–701. 10.1038/s41577-019-0185-9 31213673 10.1038/s41577-019-0185-9

[CR5] Chen J, Liu Y, Zhao Z, Qiu J (2021) Oxidative stress in the skin: impact and related protection. Int J Cosmet Sci 43(5):495–509. 10.1111/ics.12728 34312881 10.1111/ics.12728

[CR6] Fu PP, Xia Q, Sun X, Yu H (2012) Phototoxicity and environmental transformation of polycyclic aromatic hydrocarbons (PAHs)-light-induced reactive oxygen species, lipid peroxidation, and DNA damage. J Environ Sci Health C Environ Carcinog Ecotoxicol Rev 30(1):1–41. 10.1080/10590501.2012.653887 22458855 10.1080/10590501.2012.653887

[CR7] Hartwig A (2013) Polycyclic aromatic hydrocarbons (PAH), MAK value documentation 2012. Wiley-VCH, Weinheim

[CR8] Hou X, Ren C, Jin J et al (2025) Phosphoinositide signalling in cell motility and adhesion. Nat Cell Biol 27(5):736–748. 10.1038/s41556-025-01647-4 40169755 10.1038/s41556-025-01647-4

[CR9] Huang H-Y, Hsu T, Lin B-F (2019) Gamma-aminobutyric acid decreases macrophages infiltration and suppresses inflammatory responses in renal injury. J Funct Foods 60:103419. 10.1016/j.jff.2019.103419

[CR10] Jia D, Kuang Z, Wang L (2024) The role of microbial indole metabolites in tumor. Gut Microbes 16(1):2409209. 10.1080/19490976.2024.2409209 39353090 10.1080/19490976.2024.2409209 PMC11445886

[CR11] Jiang G, Kang H, Yu Y (2017) Cross-platform metabolomics investigating the intracellular metabolic alterations of HaCaT cells exposed to phenanthrene. J Chromatogr B Analyt Technol Biomed Life Sci 1060:15–21. 10.1016/j.jchromb.2017.05.023 28578192 10.1016/j.jchromb.2017.05.023

[CR12] Jin H, Lin Z, Pang T et al (2024) Effects and mechanisms of polycyclic aromatic hydrocarbons in inflammatory skin diseases. Sci Total Environ 925:171492. 10.1016/j.scitotenv.2024.171492 38458465 10.1016/j.scitotenv.2024.171492

[CR13] Kapralov AA, Yang Q, Dar HH et al (2020) Redox lipid reprogramming commands susceptibility of macrophages and microglia to ferroptotic death. Nat Chem Biol 16(3):278–290. 10.1038/s41589-019-0462-8 32080625 10.1038/s41589-019-0462-8 PMC7233108

[CR500] Kersch C, Masutin V, Alsaleh R et al (2025) Benzo[a]pyrene and UV light co-exposure: differential effects on oxidative stress and genotoxicity in human keratinocytes and ex vivo skin. Arch Toxicol. 10.1007/s00204-025-04098-w 40593241 10.1007/s00204-025-04098-w PMC12454581

[CR14] Malešević A, Tucović D, Kulaš J et al (2024) Impact of skin exposure to Benzo[a]pyrene in rat model: insights into epidermal cell function and draining lymph node cell response. Int J Mol Sci 25(16):8631 39201318 10.3390/ijms25168631 PMC11354278

[CR15] Masutin V, Kersch C, Schmitz-Spanke S (2022) A systematic review: metabolomics-based identification of altered metabolites and pathways in the skin caused by internal and external factors. Exp Dermatol 31(5):700–714. 10.1111/exd.14529 35030266 10.1111/exd.14529

[CR16] Masutin V, Kersch C, Alsaleh R, Schmitz-Spanke S (2024) Differential effects of benzo[a]pyrene exposure on glutathione and purine metabolism in keratinocytes: Dose-dependent and UV co-exposure effects. Exp Dermatol 33(3):e15044. 10.1111/exd.15044 38465766 10.1111/exd.15044

[CR17] Mirveis Z, Howe O, Cahill P, Patil N, Byrne HJ (2023) Monitoring and modelling the glutamine metabolic pathway: a review and future perspectives. Metabolomics 19(8):67. 10.1007/s11306-023-02031-9 37482587 10.1007/s11306-023-02031-9 PMC10363518

[CR18] O’Neill LA, Kishton RJ, Rathmell J (2016) A guide to immunometabolism for immunologists. Nat Rev Immunol 16(9):553–565. 10.1038/nri.2016.70 27396447 10.1038/nri.2016.70 PMC5001910

[CR19] Palsson-McDermott EM, O’Neill LAJ (2025) Gang of 3: how the Krebs cycle-linked metabolites itaconate, succinate, and fumarate regulate macrophages and inflammation. Cell Metab. 10.1016/j.cmet.2025.03.004 40169002 10.1016/j.cmet.2025.03.004

[CR20] Penning TM, Burczynski ME, Hung CF, McCoull KD, Palackal NT, Tsuruda LS (1999) Dihydrodiol dehydrogenases and polycyclic aromatic hydrocarbon activation: generation of reactive and redox active o-quinones. Chem Res Toxicol 12(1):1–18. 10.1021/tx980143n 9894013 10.1021/tx980143n

[CR21] Potratz S, Jungnickel H, Grabiger S et al (2016) Differential cellular metabolite alterations in HaCaT cells caused by exposure to the aryl hydrocarbon receptor-binding polycyclic aromatic hydrocarbons chrysene, benzo[a]pyrene and dibenzo[a,l]pyrene. Toxicol Rep 3:763–773. 10.1016/j.toxrep.2016.09.003 28959603 10.1016/j.toxrep.2016.09.003 PMC5616077

[CR22] Qiu B, Zandkarimi F, Bezjian CT et al (2024) Phospholipids with two polyunsaturated fatty acyl tails promote ferroptosis. Cell 187(5):1177–1190. 10.1016/j.cell.2024.01.030 38366593 10.1016/j.cell.2024.01.030 PMC10940216

[CR23] Randhawa M, Southall M, Samaras ST (2013) Metabolomic analysis of sun exposed skin. Mol Biosyst 9(8):2045–2050. 10.1039/c3mb25537a 23670218 10.1039/c3mb25537a

[CR24] Randhawa M, Sangar V, Tucker-Samaras S, Southall M (2014) Metabolic signature of sun exposed skin suggests catabolic pathway overweighs anabolic pathway. PLoS ONE 9(3):e90367. 10.1371/journal.pone.0090367 24603693 10.1371/journal.pone.0090367 PMC3946127

[CR25] Ranjit S, Midde NM, Sinha N et al (2016) Effect of polyaryl hydrocarbons on cytotoxicity in monocytic cells: potential role of cytochromes P450 and oxidative stress pathways. PLoS ONE 11(9):e0163827. 10.1371/journal.pone.0163827 27684561 10.1371/journal.pone.0163827 PMC5042547

[CR26] Shatova OP, Shestopalov AV (2023) Tryptophan metabolism: a new look at the role of tryptophan derivatives in the human body. Biol Bull Rev 13(2):81–91. 10.1134/s2079086423020068

[CR27] Steadman T, O’Reilly S (2025) Aberrant fumarate metabolism links interferon release in diffuse systemic sclerosis. J Dermatol Sci 117(2):30–35. 10.1016/j.jdermsci.2025.01.002 39827047 10.1016/j.jdermsci.2025.01.002

[CR28] Sukka SR, Ampomah PB, Darville LNF et al (2024) Efferocytosis drives a tryptophan metabolism pathway in macrophages to promote tissue resolution. Nat Metab 6(9):1736–1755. 10.1038/s42255-024-01115-7 39242914 10.1038/s42255-024-01115-7 PMC11734744

[CR29] Supruniuk E, Zebrowska E, Maciejczyk M, Zalewska A, Chabowski A (2023) Lipid peroxidation and sphingolipid alterations in the cerebral cortex and hypothalamus of rats fed a high-protein diet. Nutrition 107:111942. 10.1016/j.nut.2022.111942 36621260 10.1016/j.nut.2022.111942

[CR30] Tang D, Chen X, Kang R, Kroemer G (2021) Ferroptosis: molecular mechanisms and health implications. Cell Res 31(2):107–125. 10.1038/s41422-020-00441-1 33268902 10.1038/s41422-020-00441-1 PMC8026611

[CR31] Tyurina YY, St Croix CM, Watkins SC et al (2019) Redox (phospho)lipidomics of signaling in inflammation and programmed cell death. J Leukoc Biol 106(1):57–81. 10.1002/JLB.3MIR0119-004RR 31071242 10.1002/JLB.3MIR0119-004RR PMC6626990

[CR32] Vats K, Kruglov O, Mizes A et al (2021) Keratinocyte death by ferroptosis initiates skin inflammation after UVB exposure. Redox Biol 47:102143. 10.1016/j.redox.2021.102143 34592565 10.1016/j.redox.2021.102143 PMC8487085

[CR33] Wang K, Lin Y, Zhou D et al (2024) Unveiling ferroptosis: a new frontier in skin disease research. Front Immunol 15:1485523. 10.3389/fimmu.2024.1485523 39430757 10.3389/fimmu.2024.1485523 PMC11486644

[CR34] Weistenhofer W, Lutz R, Hiller J, Schmitz-Spanke S, Drexler H (2022) Syncarcinogenesis of natural UV radiation and polycyclic aromatic hydrocarbons in the development of squamous cell carcinomas of the skin? J Dtsch Dermatol Ges 20(9):1179–1186. 10.1111/ddg.14818 36075872 10.1111/ddg.14818

[CR35] Xue C, Li G, Zheng Q et al (2023) Tryptophan metabolism in health and disease. Cell Metab. 10.1016/j.cmet.2023.06.004 37352864 10.1016/j.cmet.2023.06.004

[CR36] Yang X, Wang J, Wang H, Li X, He C, Liu L (2021) Metabolomics study of fibroblasts damaged by UVB and BaP. Sci Rep 11(1):11176. 10.1038/s41598-021-90186-7 34045475 10.1038/s41598-021-90186-7 PMC8160258

[CR37] Zhu X, Meyers A, Long D et al (2019) Frontline science: Monocytes sequentially rewire metabolism and bioenergetics during an acute inflammatory response. J Leukoc Biol 105(2):215–228. 10.1002/JLB.3HI0918-373R 30633362 10.1002/JLB.3HI0918-373R PMC6466628

[CR38] Ziboh VA, Miller CC, Cho Y (2000) Metabolism of polyunsaturated fatty acids by skin epidermal enzymes: generation of antiinflammatory and antiproliferative metabolites. Am J Clin Nutr 71(1 Suppl):361S-S366. 10.1093/ajcn/71.1.361s 10617998 10.1093/ajcn/71.1.361s

