# Supplementary material for: Glutaminolysis impairment and immunometabolic dysregulation in U937 cells: Key mechanisms in occupational and environmental skin exposure to UV and benzo[a]pyrene
Source: Arch Toxicol. 2025 Aug 25;99(11):4481–92. doi: 10.1007/s00204-025-04155-4 (PMC12477086; doi:10.1007/s00204-025-04155-4)
Supplement: Supplementary file 1 — Supplementary file1 (DOCX 1456 KB) [file 204_2025_4155_MOESM1_ESM.docx]

**Supplementary Information**

Glutaminolysis impairment and immunometabolic dysregulation in U937 cells: Key mechanisms in occupational and environmental skin exposure to UV and benzo[a]pyrene

**Christian Kersch^a^, Viktor Masutin^a^, Laura Kuhlmann^a^, Rasha Alsaleh^a^,** Andrea Kaifie-Pechmann^a^, **Simone Schmitz-Spanke^a^**

**^a^Institute and Outpatient Clinic of Occupational, Social, and Environmental Medicine, Friedrich-Alexander-University of Erlangen-Nuremberg, Henkestr. 9–11, 91054 Erlangen, Germany**

[christian.kersch@fau.de](mailto:christian.kersch@fau.de)

[viktormasutin@googlemail.com](mailto:viktormasutin@googlemail.com)

laura.kuhlmann@alumni.fau.de

[rasha.alsaleh.dr@gmail.com](mailto:rasha.alsaleh.dr@gmail.com)

[andrea.kaifie-pechmann@fau.de](mailto:andrea.kaifie-pechmann@fau.de)

[simone.schmitz-spanke@fau.de](mailto:simone.schmitz-spanke@fau.de)

**Corresponding Author:**

**Simone Schmitz-Spanke**

**simone.schmitz-spanke@fau.de**

**Institute and Outpatient Clinic of Occupational, Social, and Environmental Medicine, University of Erlangen-Nuremberg, Henkestr. 9-11, 91054 Erlangen, Germany**

**Phone: +49 09131/85-22255**

**ORCID:** [**0000-0002-0416-8236**](https://orcid.org/0000-0002-0416-8236)

**Table of contens**

**[1.](#_Toc202968009)****[Supporting Methods](#_Toc202968009)** [3](#_Toc202968009)

[1.1. UV irradiation dosing 3](#_Toc202968010)

[1.2. Untargeted metabolomics (GC-MS) 3](#_Toc202968011)

*[1.2.1.](#_Toc202968012)**[Sample preparation](#_Toc202968012)* [3](#_Toc202968012)

*[1.2.2.](#_Toc202968013)**[Data processing and analysis](#_Toc202968013)* [3](#_Toc202968013)

[1.3. Targeted Metabolomics (Lipidomics) 4](#_Toc202968014)

*[1.3.1.](#_Toc202968015)**[Sample preparation](#_Toc202968015)* [4](#_Toc202968015)

*[1.3.2.](#_Toc202968016)**[LC-MS/MS Analysis](#_Toc202968016)* [5](#_Toc202968016)

[1.4. Toxicological assays 6](#_Toc202968017)

*[1.4.1.](#_Toc202968018)**[Determination of oxidative stress](#_Toc202968018)* [6](#_Toc202968018)

*[1.4.2.](#_Toc202968019)**[Determination of cell viability and mitochondrial function](#_Toc202968019)* [6](#_Toc202968019)

*[1.4.3.](#_Toc202968020)**[Determination of DNA damage](#_Toc202968020)* [7](#_Toc202968020)

**[2.](#_Toc202968021)****[Supporting tables](#_Toc202968021)** [8](#_Toc202968021)

[2.1. Untargeted Metabolomics 8](#_Toc202968022)

[2.1.1. Comparison of groups exposed to 4 µM B[a]P (high) and 4 µM B[a]P +UV versus control conditions 10](#_Toc202968023)

[2.1.2. Comparing the impact of combined B[a]P+UV exposure to control conditions across a range of B[a]P concentrations 14](#_Toc202968024)

[2.2. Targeted Lipidomics 17](#_Toc202968025)

[2.3. Toxicological Assays 22](#_Toc202968026)

**[3.](#_Toc202968027)****[Supporting figures](#_Toc202968027)** [23](#_Toc202968027)

[3.1. Untargeted Metabolomics 23](#_Toc202968028)

*[3.1.1.](#_Toc202968029)**[Comparison of groups exposed to 4 µM B[a]P (high) and 4 µM B[a]P +UV versus control conditions](#_Toc202968029)* [23](#_Toc202968029)

*[3.1.2.](#_Toc202968030)**[Comparing the impact of combined B[a]P+UV exposure to control conditions across a range of B[a]P concentrations](#_Toc202968030)* [28](#_Toc202968030)

[3.2. Targeted Lipidomics 30](#_Toc202968031)

# **Supporting Methods**

## UV irradiation dosing

The detailed derivation of the base UV dose, calculated from meteorological data for environmentally relevant sunlight exposure, is comprehensively described in the Supplementary Data in our related work (Kersch et al. 2025).For the U937 cells, we specifically applied a UVA dose of 1.7 J/cm². This dose was carefully selected to simulate a physiologically relevant exposure for immune cells located in the deeper dermal layers. This involved using only UVA (given its greater skin penetration compared to UVB (Meinhardt et al. 2008) and further reducing the dose by half to account for the attenuation of UV radiation during its passage through the epidermal layer before reaching dermal cells.

## Untargeted metabolomics (GC-MS)

### *1.2.1. Sample preparation*

Following exposure, cells were washed twice with 0.9% saline and stored at -80°C. Subsequently, 150 μL of cold methanol was added to each well, and cells were scraped and transferred to 2 mL tubes. 450 μL of methyl tert-butyl ether (MTBE) was added to each tube, and cells were homogenized with glass beads for 1 minute at 4800 rpm. Phase separation was achieved by adding 200 μL of saturated sodium chloride solution, followed by vortexing for 3 minutes and centrifugation at 18,000 x g for 5 minutes at 4°C. This phase separation step was repeated with 400 μL of MTBE:methanol (10:3, v/v). 500 μL of each organic and aqueous phase was collected separately in 2 mL tubes and dried under vacuum in a SpeedVac concentrator for 3 hours. The dried aqueous fraction was incubated with 50 μL of 20 mg/mL methoxyamine hydrochloride in pyridine at 50°C for 1 hour, followed by derivatization with 25 μL of N-methyl-N-(trimethylsilyl)-trifluoroacetamide (MSTFA) containing 1% trimethylchlorosilane (TMCS) at 40°C for 1 hour. Lipids in the dried organic fraction were extracted twice with 300 μL of hexane:water (1:1, v/v), the organic phase was dried, dissolved in 50µL pyridine and derivatized with 25 μL of MSTFA containing 1% TMCS at 40°C for 1 hour. Finally, the derivatized aqueous and organic fractions were combined and analyzed by gas chromatography-mass spectrometry (GC-MS).

### *1.2.2. Data processing and analysis*

Peak deconvolution and identification of metabolic signatures from raw mass data (GC-MS) was conducted using R studio and annotated Golm metabolome database (GMD) package (http://gmd.mpimp-golm.mpg.de/download/). Detected metabolic features were identified by erah (R package) using an annotated GMD.11 All features with a match factor (MF) >90% were included for further statistical analysis. The generated metabolic data were normalized using % area normalization. In this case, the sum of all peak areas in the chromatogram was assumed as 100%. Afterwards, the individual peak areas were calculated according to this 100% area. The data were pretreated by log10 transformation to reduce heteroscedasticity and multiplicative effects, and Pareto scaling was performed to minimize the relative importance of large values.

Untargeted metabolomics data were processed and analyzed using MetaboAnalyst 6.0. Multivariate analysis of metabolite abundance ratios (principal component analysis (PCA), orthogonal partial least squares-discriminant analysis (OPLS-DA), variable importance in projection (VIP)) as well as metabolic enrichment and pathway analysis were performed by uploading normalized data to MetaboAnalyst 6.0.

For univariate statistical comparisons, specific criteria were applied based on the type of analysis. For two-group comparisons (t-tests), metabolites were considered altered if their raw p-value was below 0.1 and met a fold-change threshold of 1.5. For multiple-group comparisons using one-way ANOVA, the overall significance of metabolites was determined with an FDR-adjusted p-value cutoff of 0.1. For post-hoc analyses following parametric ANOVA (e.g., Fisher's LSD), a raw p-value cutoff of 0.05 was applied for specific group differences.

For the initial identification of individual differentially regulated metabolites for visualization in volcano plots (as presented in Supplementary Figures) and their listing in Supplementary Tables S2-S4, metabolites were considered to be altered if they met the aforementioned criteria. These criteria were applied following the specific settings within MetaboAnalyst 6.0.

For metabolic pathway analysis, the Hypergeometric Test was used as the enrichment method, and appropriate multiple testing corrections (e.g., False Discovery Rate - FDR using the Benjamini-Hochberg method) were consistently applied, with FDR values for identified pathways reported in the corresponding supplementary tables.

Data visualization was performed using SankeyMATIC software to create a Sankey diagram.

## 1.3. Targeted Metabolomics (Lipidomics)

### *1.3.1. Sample preparation*

Following exposure, cells were washed twice with 0.9% saline and stored at -80°C to quench metabolism. For lysis, the cells were thawed, centrifuged (5000 rpm, 5 min), sonicated for 3 minutes, and then frozen again to further disrupt the cell structure. This freeze-thaw-centrifugation-sonication cycle was repeated once to ensure thorough cell lysis. Subsequently, the samples were thawed, and 1.2 mL of methanol was added to precipitate proteins and extract polar metabolites. After vortexing to ensure proper mixing, the samples were centrifuged (12000 rpm, 5 min), and 1 mL of the supernatant, containing the extracted metabolites, was collected and dried in a vacuum centrifuge. The dried residue was then resuspended in 25 µL of solvent (50% chloroform, 50% methanol) to aid in the extraction of a broader range of metabolites. This mixture was vortexed, centrifuged (15000 rpm, 5 min), and sonicated for 3 minutes to ensure complete dissolution. Finally, 225 µL of a second solvent (5% chloroform, 5% methanol, 90% acetonitrile) was added to adjust the solvent composition for subsequent analysis, and the mixture was incubated on an orbital shaker for 10 minutes before a final centrifugation step. 100 µL of the resulting supernatant was transferred to a glass vial for analysis.

Prior to performing targeted lipidomics, a thorough review of the literature was conducted to identify and select lipids of particular interest for this study (Cai et al. 2009; Hsu and Turk 2001; Masutin et al. 2022; Milne et al. 2006; Pi et al. 2016).

### *1.3.2. LC-MS/MS Analysis*

Liquid chromatography was performed on a Waters ACQUITY® UPLC H-Class system equipped with a quaternary pump (Waters ACQ H-Class QSM Plus), a temperature-controlled autosampler (Waters ACQ H-Class FTN-H Plus), and a column manager (ACQUITY® UHPLC CM-A). The separation was achieved using an ACQUITY HSS T3 column (2.1 x 100 mm, 1.8 µm) from Waters GmbH (Eschborn, Germany), maintained at a column temperature of 65°C and a flow rate of 500 µL/min. The total runtime was 20 minutes. The mobile phases consisted of: (A) acetonitrile/water (40:60, v/v) with 10 mM ammonium formate at pH 5.0, and (B) acetonitrile/isopropanol (10:90, v/v) with 10 mM ammonium formate at pH 5. The gradient elution program was as follows: 100 % A for one minute. Followed by two linear increases, one to 40 % B over four minutes, the other to 100 % B over 10 minutes. After two minutes at 100 % B it was switched back to 40 % B for three minutes. The mass spectrometer was operated with a capillary voltage of 3.2 kV, a source temperature of 150°C, and multiple reaction monitoring (MRM) as the acquisition mode. The Waters Xevo® TQ-XS mass spectrometer with an electrospray source operated in alternating positive and negative ionization mode.

**L**iquid chromatography gradient program.

| **min** | **A** | **B** | **Water** | **Isopropanol** | **Acetonitrile** |
| --- | --- | --- | --- | --- | --- |
| 0 | 1 | 0 | 60% | 0% | 40% |
| 1 | 1 | 0 | 60% | 0% | 40% |
| 5 | 0.6 | 0.4 | 36% | 36% | 28% |
| 15 | 0 | 1 | 0% | 90% | 10% |
| 17 | 0 | 1 | 0% | 90% | 10% |
| 17.1 | 0.6 | 0.4 | 36% | 36% | 28% |
| 20 | 0.6 | 0.4 | 36% | 36% | 28% |

## 1.4. Toxicological assays

### *1.4.1. Determination of oxidative stress*

Oxidative stress was assessed by evaluating reactive oxygen species (ROS) production, glutathione redox status (GSH/GSSG ratio), lipid peroxidation, and activity of NQO1 (NAD(P)H dehydrogenase (quinone 1)), a phase II antioxidant enzyme.

Intracellular ROS levels were determined using the fluorescent probe 2′,7′-dichlorodihydrofluorescein diacetate (H2DCF-DA) (Thermo Fisher Scientific, Kandel, Germany). After washing with PBS, cells were incubated with 20 μM H2DCF-DA for 30 minutes at 37 °C in a humidified atmosphere containing 5% CO2. Fluorescence was measured at excitation/emission wavelengths of 485/535 nm.

For measuring the ratio of reduced glutathione (GSH) to oxidized glutathione (GSSG), cells were washed twice with PBS and stored at -80 °C. Upon thawing, a reaction mixture containing PBS, EDTA, DTNB, GSSG reductase, and Triton X-100 was added to each well. After a brief incubation, NADPH was added to initiate the reaction, and the absorbance was measured at 415 nm. To determine GSSG levels, an inhibitor solution containing 1-methyl-2-vinylpyridinium triflate and Triton X-100 was added prior to the NADPH addition, and the procedure was repeated as described for GSH.

Lipid peroxidation was assessed by measuring malondialdehyde (MDA) formation using the thiobarbituric acid reactive substances (TBARS) assay. Cells were washed with PBS and incubated with 1-methyl-2-phenylindole in the presence of HCl. After incubation at 45 °C, the absorbance of the resulting chromophore was measured at 586 nm.

NQO1 activity was measured by a colorimetric assay. After exposure, cells were washed with PBS and stored at -80 °C. Upon thawing, a reaction mixture containing Tris buffer, NADH, and Triton X-100 was added to each well. Following incubation, dichlorophenolindophenol was added as a substrate, and the decrease in absorbance at 600 nm was monitored.

### *1.4.2. Determination of cell viability and mitochondrial function*

Cell viability was assessed by measuring lactate dehydrogenase (LDH) release, indicative of membrane damage. Mitochondrial function was evaluated through MTT ((3-(4,5-dimethylthiazol-2-yl)-2,5-diphenyltetrazolium bromide) reduction and mitochondrial membrane potential (MMP) analysis.

LDH activity in the culture medium was determined by measuring the reduction of iodonitrotetrazolium chloride (INT) to formazan. 50 µL of culture supernatant were incubated with a reaction mixture containing 200 mM Tris-HCl (pH 8.0), 50 mM sodium lactate, 30 mM phenazine methosulfate, 65 mM INT in DMSO, and 5.5 mM NAD+ for 5 minutes at room temperature. The absorbance of the resulting formazan product was measured at 490 nm

Cellular metabolic activity was assessed by measuring the conversion of MTT to formazan. MTT was added to the cells two hours prior to the end of the exposure period at a final concentration of 0.13 mM. After incubation, the supernatant was removed and replaced with 100 μL of a solubilization solution containing 0.6% glacial acetic acid and 10% SDS in DMSO. Following a ten-minute incubation with agitation, the absorbance of the solubilized formazan product was measured at 600 nm.

MMP was assessed using the fluorescent probe rhodamine 123. After the exposure period, cells were washed with PBS. Subsequently, 100 μL of a solution containing 1 μM propidium iodide and 1 μM rhodamine 123 was added to each well and incubated for 30 minutes at 37°C in a 5% CO2 atmosphere. Propidium iodide was used to quantify the number of dead cells. Following two washes with PBS, fluorescence measurements were acquired at excitation/emission wavelengths of 485/535 nm for rhodamine 123 and 535/617 nm for PI.

### *1.4.3. Determination of DNA damage*

The alkaline comet assay was performed to assess DNA damage. Following exposure, cells were washed twice with PBS and trypsinized. A cell suspension was prepared at a concentration of 250,000 cells/mL, and 20 μL of this suspension was mixed with 140 μL of 1% low-melting point agarose at 38°C. Twenty microliters of this mixture was then pipetted onto precoated slides. Positive control cells were treated with 50 μM hydrogen peroxide (H2O2) for 5 minutes. Slides were lysed in a solution containing 2.5 M NaCl, 0.1 M EDTA, 10 mM Tris, and 1% Triton X-100 on ice for one hour in the dark. Subsequently, slides were immersed in alkaline electrophoresis buffer (0.3 M NaOH, 1 mM EDTA) at 4°C for 40 minutes. Electrophoresis was then conducted at 4°C for 25 minutes at a voltage gradient of 1 V/cm and a constant current of 300 mA. Following electrophoresis, gels were washed twice with 0.04 M Tris for 5 minutes and once with water for 3 minutes before being allowed to air-dry in the dark. Gels were stained with SYBR® Green nucleic acid stain (1:10,000 dilution) and visualized using a Leica microscope equipped with a charge-coupled device camera. The olive tail moment (OTM) of 100 comets per sample was automatically calculated using Comet Assay IV software (Perspective Instruments, UK)

# **Supporting tables**

## Untargeted Metabolomics

**Table S 1:** List of all metabolites identified by GC-MS with match factors >0.9 according to the Golm Metabolome Database (GMD)

| **No.** | **Metabolites** |
| --- | --- |
| 1 | ƴ-Linolenic acid |
| 2 | Linoleic Acid |
| 3 | 1,2,4-Butanetriol |
| 4 | 1,3-Di-tert-butylbenzene |
| 5 | Inositol 2-phosphate |
| 6 | 2-Hydroxybutyric acid |
| 7 | 3-Sulfinoalanine |
| 8 | 4-Hydroxybutyric acid |
| 9 | 5-Hydroxy-L-tryptophan |
| 10 | 5'-Methylthioadenosine |
| 11 | 6-Kestose |
| 12 | Adenine |
| 13 | Adenosine monophosphate (AMP) |
| 14 | Alanine |
| 15 | Alanylalanine |
| 16 | Asparagine |
| 17 | Aspartate |
| 18 | Benzoic acid |
| 19 | Cadaverine |
| 20 | Cholesterol |
| 21 | Cinnamic acid |
| 22 | Citrate |
| 23 | Malate |
| 24 | D-Glucose |
| 25 | Pipecolic acid |
| 26 | Docosahexaenoic acid |
| 27 | Eicosanoic acid |
| 28 | Eicosenoic acid |
| 29 | Oleic acid |
| 30 | Fumarate |
| 31 | GABA |
| 32 | Galactose |
| 33 | Glucose-1-phosphate |
| 34 | Glutamine |
| 35 | Glutaric acid |
| 36 | Glycerol |
| 37 | Glycine |
| 38 | Guanosine-5'-monophosphate (GMP) |
| 39 | Histamine |
| 40 | Hydroxyphenyllactic acid |
| 41 | Hypoxanthine |
| 42 | Indoleacetaldehyde |
| 43 | Indolelactic acid |
| 44 | Inositol |
| 45 | Isoleucine |
| 46 | Isovaleric acid |
| 47 | Lactate |
| 48 | Lauric acid |
| 49 | Leucine |
| 50 | Glutamate |
| 51 | Homocitrulline |
| 52 | Norleucine |
| 53 | Ornithine |
| 54 | Pyroglutamic acid |
| 55 | Serine |
| 56 | Sorbose |
| 57 | Threonine |
| 58 | Tryptophan |
| 59 | Mandelic acid |
| 60 | Methane |
| 61 | Myristamide |
| 62 | Myristic acid |
| 63 | N-Acetyl-L-aspartic acid |
| 64 | N-Acetylserine |
| 65 | N-α-Acetyl-L-Lysine |
| 66 | Niacinamide |
| 67 | Octanoic acid |
| 68 | Oleamide |
| 69 | Palmitic acid |
| 70 | Palmitic amide |
| 71 | Pelargonic acid |
| 72 | Pentadecanoic acid |
| 73 | Phosphoric acid |
| 74 | Phytosphingosine |
| 75 | Proline |
| 76 | Pyrophosphate |
| 77 | Serotonin |
| 78 | Sphingosine |
| 79 | Stearic acid |
| 80 | Stearidonic acid |
| 81 | Succinic acid |
| 82 | Thymine |
| 83 | Tyrosine |
| 84 | Urea |
| 85 | Valine |

### Comparison of groups exposed to 4 µM B[a]P (high) and 4 µM B[a]P +UV versus control conditions

**Table S2:** Metabolites altered in U937 cells exposed to **UV** irradiation (n=3) identified by volcano plot analysis (raw p-value < 0.1; FC > 1.5) in MetaboAnalyst 6.0.

| **No.** | **Metabolites** | **FC** | **log2(FC)** | **raw.pval** | **-log10(p)** |
| --- | --- | --- | --- | --- | --- |
| 1 | Glucose-1-phosphate | 2.01 | 1.0072 | 0.088547 | 1.0528 |
| 2 | Glutamate | 1.9539 | 0.96633 | 0.094561 | 1.0243 |
| 3 | Mandelic acid | 2.195 | 1.1342 | 0.09913 | 1.0038 |

**Table S3:** Metabolites altered in U937 cells exposed to **4 µM B[a]P** (n=3) identified by volcano plot analysis (raw p-value < 0.1; Fold Change > 1.5) in MetaboAnalyst 6.0.

| **No.** | **Metabolites** | **FC** | **log2(FC)** | **raw.pval** | **-log10(p)** |
| --- | --- | --- | --- | --- | --- |
| 1 | Palmitic amide | 1.567 | 0.64799 | 0.01118 | 1.9516 |
| 2 | Indoleacetaldehyde | 0.65331 | -0.61415 | 0.012096 | 1.9173 |
| 3 | 5-Hydroxy-L-tryptophan | 0.65751 | -0.60491 | 0.033361 | 1.4768 |
| 4 | Fumarate | 1.5218 | 0.60582 | 0.036656 | 1.4359 |
| 5 | 3-Sulfinoalanine | 1.5121 | 0.59653 | 0.047513 | 1.3232 |
| 6 | Glutamate | 2.1978 | 1.1361 | 0.081119 | 1.0909 |

**Table S4:** Metabolites altered in U937 cells exposed to **4 µM B[a]P and UV irradiation** (n=3) identified by volcano plot analysis (raw p-value < 0.1; Fold Change > 1.5) in MetaboAnalyst 6.0.

| **No.** | **Metabolites** | **FC** | **log2(FC)** | **raw.pval** | **-log10(p)** |
| --- | --- | --- | --- | --- | --- |
| 1 | 5'-Methylthioadenosine | 0.013823 | -6.1768 | 1.90E-05 | 4.7221 |
| 2 | Glutaric acid | 0.056864 | -4.1363 | 2.06E-05 | 4.6869 |
| 3 | N-Acetyl-L-aspartic acid | 0.044566 | -4.4879 | 3.78E-05 | 4.4229 |
| 4 | Niacinamide | 0.025849 | -5.2737 | 3.96E-05 | 4.402 |
| 5 | Fumarate | 0.034568 | -4.8544 | 4.34E-05 | 4.3621 |
| 6 | Malate | 0.014001 | -6.1583 | 6.46E-05 | 4.1899 |
| 7 | Alanylalanine | 0.042807 | -4.546 | 0.00010173 | 3.9926 |
| 8 | Adenosine monophosphate | 0.0014519 | -9.4278 | 0.00017955 | 3.7458 |
| 9 | Citrate | 0.013378 | -6.224 | 0.00018779 | 3.7263 |
| 10 | Glutamine | 0.056072 | -4.1566 | 0.00019131 | 3.7183 |
| 11 | Inositol | 0.099563 | -3.3282 | 0.00024823 | 3.6051 |
| 12 | Indolelactic acid | 0.071133 | -3.8133 | 0.00029877 | 3.5247 |
| 13 | Proline | 0.027503 | -5.1843 | 0.00038621 | 3.4132 |
| 14 | Glutamate | 0.008845 | -6.8209 | 0.00045183 | 3.345 |
| 15 | ƴ-Linolenic acid | 18.467 | 4.2069 | 0.00067789 | 3.1688 |
| 16 | Pentadecanoic acid | 0.27869 | -1.8433 | 0.00087115 | 3.0599 |
| 17 | Inositol 2-phosphate | 0.20178 | -2.3092 | 0.00095619 | 3.0195 |
| 18 | Glycine | 0.053472 | -4.2251 | 0.0010685 | 2.9712 |
| 19 | Galactose | 43.054 | 5.4281 | 0.0012646 | 2.898 |
| 20 | Alanine | 0.1397 | -2.8395 | 0.00198 | 2.7033 |
| 21 | Pyroglutamic acid | 0.11582 | -3.1101 | 0.0020834 | 2.6812 |
| 22 | Aspartate | 0.068678 | -3.864 | 0.0021658 | 2.6644 |
| 23 | Lactate | 0.044788 | -4.4807 | 0.0036794 | 2.4342 |
| 24 | Isoleucine | 0.14106 | -2.8256 | 0.0038761 | 2.4116 |
| 25 | Valine | 0.11119 | -3.1689 | 0.0041015 | 2.3871 |
| 26 | Asparagine | 0.056598 | -4.1431 | 0.0085627 | 2.0674 |
| 27 | Linoleic Acid | 0.36335 | -1.4606 | 0.010452 | 1.9808 |
| 28 | Eicosenoic acid | 0.38316 | -1.384 | 0.012358 | 1.9081 |
| 29 | GABA | 0.042973 | -4.5404 | 0.016954 | 1.7707 |
| 30 | Serotonin | 0.33016 | -1.5988 | 0.017615 | 1.7541 |
| 31 | Oleic acid | 0.39624 | -1.3355 | 0.019878 | 1.7016 |
| 32 | Norleucine | 0.16196 | -2.6263 | 0.022189 | 1.6539 |
| 33 | Ornithine | 0.26935 | -1.8924 | 0.023762 | 1.6241 |
| 34 | 2-Hydroxybutyric acid | 0.34146 | -1.5502 | 0.026569 | 1.5756 |
| 35 | Hypoxanthine | 0.35364 | -1.4996 | 0.033843 | 1.4705 |
| 36 | Cholesterol | 0.4554 | -1.1348 | 0.034247 | 1.4654 |
| 37 | Guanosine-5'-monophosphate | 0.18969 | -2.3983 | 0.03882 | 1.4109 |
| 38 | Thymine | 0.37602 | -1.4111 | 0.049052 | 1.3093 |
| 39 | Glucose-1-phosphate | 0.35477 | -1.495 | 0.053084 | 1.275 |
| 40 | Mandelic acid | 2.2668 | 1.1806 | 0.054271 | 1.2654 |
| 41 | Leucine | 0.073924 | -3.7578 | 0.065272 | 1.1853 |
| 42 | Phytosphingosine | 5.9564 | 2.5745 | 0.072494 | 1.1397 |
| 43 | Hydroxyphenyllactic acid | 0.23199 | -2.1079 | 0.087787 | 1.0566 |
| 44 | Methane | 0.13316 | -2.9088 | 0.092594 | 1.0334 |
| 45 | Myristic acid | 0.18663 | -2.4218 | 0.094608 | 1.0241 |
| 46 | Docosahexaenoic acid | 4.1045 | 2.0372 | 0.096563 | 1.0152 |

**Table S5:** Significantly altered pathways following exposure to **UV** irradiation (p< 0.05) identified in U937 cells (n = 3) using pathway analysis in MetaboAnalyst 6.0

| Pathway | Total Cmpd | Hits | Raw p | -LOG10(p) | Holm adjust | FDR | Impact | Pathway coverage [%] |
| --- | --- | --- | --- | --- | --- | --- | --- | --- |
| Inositol phosphate metabolism | 30 | 1 | 0.043 | 1.36 | 1 | 0.59 | 0.123 | 3 |
| Ascorbate and aldarate metabolism | 9 | 1 | 0.043 | 1.36 | 1 | 0.59 | 0 | 11 |

**Table S6:** Significantly altered pathway following exposure to **B[a]P** (p< 0.05) identified in U937 cells (n = 3) using pathway analysis in MetaboAnalyst 6.0

| Pathway | Total Cmpd | Hits | Raw p | -LOG10(p) | Holm adjust | FDR | Impact | Pathway coverage [%] |
| --- | --- | --- | --- | --- | --- | --- | --- | --- |
| Taurine and hypotaurine metabolism | 8 | 1 | 0.047 | 1.32 | 1 | 0.73 | 0.17143 | 13 |

**Table S7:** Significantly altered pathways following exposure to **B[a]P and UV** (p< 0.05) identified in U937 cells (n = 3) using pathway analysis in MetaboAnalyst 6.0

|  | Total Cmpd | Hits | Raw p | -LOG10(p) | Holm adjust | FDR | Impact | Pathway coverage [%] |
| --- | --- | --- | --- | --- | --- | --- | --- | --- |
| Galactose metabolism | 27 | 5 | 7.52E-07 | 6.12 | 0.00004 | 0.00004 | 0.40 | 19 |
| Glyoxylate and dicarboxylate metabolism | 32 | 5 | 1.27E-05 | 4.90 | 0.00062 | 0.00028 | 0.18 | 16 |
| Alanine, aspartate and glutamate metabolism | 28 | 10 | 1.69E-05 | 4.77 | 0.00081 | 0.00028 | 0.71 | 36 |
| Porphyrin metabolism | 31 | 2 | 2.65E-05 | 4.58 | 0.00125 | 0.00033 | 0.00 | 6 |
| Pyrimidine metabolism | 39 | 2 | 3.57E-05 | 4.45 | 0.00164 | 0.00036 | 0.04 | 5 |
| Glutathione metabolism | 28 | 5 | 5.21E-05 | 4.28 | 0.00235 | 0.00043 | 0.12 | 18 |
| Citrate cycle (TCA cycle) | 20 | 3 | 9.19E-05 | 4.04 | 0.00404 | 0.00066 | 0.15 | 15 |
| Nitrogen metabolism | 6 | 2 | 1.47E-04 | 3.83 | 0.00632 | 0.00092 | 0.00 | 33 |
| Arginine biosynthesis | 14 | 6 | 2.27E-04 | 3.64 | 0.00952 | 0.00103 | 0.18 | 43 |
| Purine metabolism | 70 | 6 | 2.43E-04 | 3.61 | 0.00998 | 0.00103 | 0.17 | 9 |
| Inositol phosphate metabolism | 30 | 1 | 2.48E-04 | 3.61 | 0.00998 | 0.00103 | 0.13 | 3 |
| Ascorbate and aldarate metabolism | 9 | 1 | 2.48E-04 | 3.61 | 0.00998 | 0.00103 | 0.00 | 11 |
| Nicotinate and nicotinamide metabolism | 15 | 2 | 2.72E-04 | 3.57 | 0.01034 | 0.00105 | 0.19 | 13 |
| Glycolysis / Gluconeogenesis | 26 | 2 | 3.56E-04 | 3.45 | 0.01318 | 0.00127 | 0.00 | 8 |
| Arginine and proline metabolism | 36 | 4 | 3.86E-04 | 3.41 | 0.01389 | 0.00129 | 0.23 | 11 |
| Pyruvate metabolism | 23 | 2 | 4.72E-04 | 3.33 | 0.01653 | 0.00148 | 0.00 | 9 |
| Cysteine and methionine metabolism | 33 | 3 | 6.10E-04 | 3.21 | 0.02073 | 0.00179 | 0.06 | 9 |
| Histidine metabolism | 16 | 3 | 7.34E-04 | 3.13 | 0.02422 | 0.00204 | 0.19 | 19 |
| Lipoic acid metabolism | 28 | 1 | 1.07E-03 | 2.97 | 0.03419 | 0.00281 | 0.00 | 4 |
| Pantothenate and CoA biosynthesis | 20 | 2 | 1.63E-03 | 2.79 | 0.05048 | 0.00407 | 0.00 | 10 |
| Primary bile acid biosynthesis | 46 | 2 | 1.96E-03 | 2.71 | 0.05876 | 0.00450 | 0.06 | 4 |
| Selenocompound metabolism | 20 | 1 | 1.98E-03 | 2.70 | 0.05876 | 0.00450 | 0.00 | 5 |
| beta-Alanine metabolism | 21 | 1 | 2.17E-03 | 2.66 | 0.06064 | 0.00464 | 0.00 | 5 |
| Tyrosine metabolism | 42 | 2 | 2.28E-03 | 2.64 | 0.06149 | 0.00464 | 0.16 | 5 |
| Butanoate metabolism | 15 | 3 | 2.32E-03 | 2.63 | 0.06149 | 0.00464 | 0.03 | 20 |
| Biosynthesis of unsaturated fatty acids | 36 | 6 | 4.17E-03 | 2.38 | 0.10420 | 0.00802 | 0.00 | 17 |
| Valine, leucine and isoleucine degradation | 40 | 3 | 6.86E-03 | 2.16 | 0.16469 | 0.01271 | 0.00 | 8 |
| Valine, leucine and isoleucine biosynthesis | 8 | 4 | 8.81E-03 | 2.05 | 0.20272 | 0.01574 | 0.00 | 50 |
| Propanoate metabolism | 22 | 2 | 9.39E-03 | 2.03 | 0.20660 | 0.01619 | 0.00 | 9 |
| Linoleic acid metabolism | 5 | 1 | 1.05E-02 | 1.98 | 0.21949 | 0.01732 | 1.00 | 20 |
| Glycine, serine and threonine metabolism | 33 | 3 | 1.07E-02 | 1.97 | 0.21949 | 0.01732 | 0.47 | 9 |
| Steroid biosynthesis | 41 | 1 | 3.42E-02 | 1.47 | 0.65068 | 0.05189 | 0.03 | 2 |
| Steroid hormone biosynthesis | 87 | 1 | 3.42E-02 | 1.47 | 0.65068 | 0.05189 | 0.01 | 1 |

### Comparing the impact of combined B[a]P+UV exposure to control conditions across a range of B[a]P concentrations

**Table S8:** Significantly altered metabolites after exposure to low (**0.04 nM**), medium (**4 nM**) B[a]P and high (**4 µM**) B[a]P combined with **UV** irradiation identified in U937 cells (n = 3) using MetaboAnalyst 6.0 (ANOVA & post-hoc Tests (FDR, q = 0.1; LSD, α = 0.05):

|  | f.value | p.value | -LOG10(p) | FDR | Fisher's LSD |  |
| --- | --- | --- | --- | --- | --- | --- |
| Malate | 75.3 | 3.32E-06 | 5.48 | 0.0003 | Control - high B[a]P+UV | |
| Galactose | 42.962 | 2.80E-05 | 4.55 | 0.0012 | high B[a]P+UV - Control | |
| Inositol | 33.766 | 6.85E-05 | 4.16 | 0.0019 | Control - high B[a]P+UV | |
| Glutaric acid | 29.427 | 1.13E-04 | 3.95 | 0.0024 | Control - high B[a]P+UV | |
| Glutamine | 23.016 | 2.74E-04 | 3.56 | 0.0039 | Control - high B[a]P+UV | |
| AMP | 22.943 | 2.77E-04 | 3.56 | 0.0039 | Control - high B[a]P+UV | |
| ƴ-Linolenic acid | 19.469 | 4.93E-04 | 3.31 | 0.0060 | high B[a]P+UV - Control | |
| Niacinamide | 12.086 | 2.43E-03 | 2.61 | 0.0258 | Control - high B[a]P+UV | |
| Phytosphingosine | 10.931 | 3.34E-03 | 2.48 | 0.0316 | high B[a]P+UV - Control | |
| GABA | 10.065 | 4.32E-03 | 2.36 | 0.0367 | Control - high B[a]P+UV | |
| 5'-Methylthioadenosine | 8.2575 | 7.84E-03 | 2.11 | 0.0600 | Control - high B[a]P+UV | |
| Pyroglutamic acid | 7.8409 | 9.11E-03 | 2.04 | 0.0600 | Control - high B[a]P+UV | |
| Citrate | 7.8224 | 9.17E-03 | 2.04 | 0.0600 | Control - high B[a]P+UV | |
| Alanine | 7.4702 | 1.05E-02 | 1.98 | 0.0636 | Control - high B[a]P+UV | |
| Thymine | 7.2802 | 1.13E-02 | 1.95 | 0.0638 | Control - high B[a]P+UV | |
| Indolelactic acid | 6.9925 | 1.26E-02 | 1.90 | 0.0641 | Control - high B[a]P+UV | |
| Asparagine | 6.9506 | 1.28E-02 | 1.89 | 0.0641 | Control - high B[a]P+UV | |
| 3-Sulfinoalanine | 6.1748 | 1.77E-02 | 1.75 | 0.0806 | low B[a]P+UV - high B[a]P+UV | |
| Proline | 6.1368 | 1.80E-02 | 1.74 | 0.0806 | Control - high B[a]P+UV | |
| Inositol 2-phosphate | 5.9201 | 1.98E-02 | 1.70 | 0.0816 | Control - high B[a]P+UV | |
| Hydroxyphenyllactic acid | 5.8839 | 2.02E-02 | 1.70 | 0.0816 | Control - high B[a]P+UV | |
| 2-Hydroxybutyric acid | 5.6084 | 2.29E-02 | 1.64 | 0.0850 | Control - high B[a]P+UV | |
| Hypoxanthine | 5.5255 | 2.37E-02 | 1.62 | 0.0850 | Control - high B[a]P+UV | |
| Docosahexaenoic acid | 5.503 | 2.40E-02 | 1.62 | 0.0850 | high B[a]P+UV - Control | |
| Fumarate | 5.2967 | 2.65E-02 | 1.58 | 0.0900 | Control - high B[a]P+UV | |

**Table S9:** Significantly altered metabolites (FC < 0.666 or FC > 1.5) identified in U937 cells exposed to combined **low (0.04nM) B[a]P+UV** (n = 3) using volcano plot analysis in MetaboAnalyst 6.0.

| **No.** | **Metabolites** | **FC** | **log2(FC)** | **raw.pval** | **-log10(p)** |
| --- | --- | --- | --- | --- | --- |
| 1 | Glycerol | 0.46782 | -1.096 | 0.021047 | 1.6768 |
| 2 | Lactate | 0.47298 | -1.0801 | 0.058219 | 1.2349 |
| 3 | Eicosenoic acid | 0.57948 | -0.78717 | 0.097129 | 1.0127 |
| 4 | 2-Hydroxybutyric acid | 0.61392 | -0.70388 | 0.042775 | 1.3688 |
| 5 | Glucose-1-phosphate | 2.0558 | 1.0397 | 0.010868 | 1.9639 |

**Table S10:** Significantly altered metabolites (FC < 0.666 or FC > 1.5) identified in U937 cells exposed to combined **high (4µM) B[a]P+UV** (n = 3) using volcano plot analysis in MetaboAnalyst 6.0.

| **No.** | **Metabolites** | **FC** | **log2(FC)** | **raw.pval** | **-log10(p)** |
| --- | --- | --- | --- | --- | --- |
| 1 | AMP | 0.0014519 | -9.4278 | 0.00017955 | 3.7458 |
| 2 | Glutamate | 0.008845 | -6.8209 | 0.00045183 | 3.345 |
| 3 | Citrate | 0.013378 | -6.224 | 0.00018779 | 3.7263 |
| 4 | 5'-Methylthioadenosine | 0.013823 | -6.1768 | 1.90E-05 | 4.7221 |
| 5 | Malate | 0.014001 | -6.1583 | 6.46E-05 | 4.1899 |
| 6 | Niacinamide | 0.025849 | -5.2737 | 3.96E-05 | 4.402 |
| 7 | Proline | 0.027503 | -5.1843 | 0.00038621 | 3.4132 |
| 8 | Fumarate | 0.034568 | -4.8544 | 4.34E-05 | 4.3621 |
| 9 | Alanylalanine | 0.042807 | -4.546 | 0.00010173 | 3.9926 |
| 10 | GABA | 0.042973 | -4.5404 | 0.016954 | 1.7707 |
| 11 | N-Acetyl-L-aspartic acid | 0.044566 | -4.4879 | 3.78E-05 | 4.4229 |
| 12 | Lactate | 0.044788 | -4.4807 | 0.0036794 | 2.4342 |
| 13 | Glycine | 0.053472 | -4.2251 | 0.0010685 | 2.9712 |
| 14 | Glutamine | 0.056072 | -4.1566 | 0.00019131 | 3.7183 |
| 15 | Asparagine | 0.056598 | -4.1431 | 0.0085627 | 2.0674 |
| 16 | Glutaric acid | 0.056864 | -4.1363 | 2.06E-05 | 4.6869 |
| 17 | Aspartate | 0.068678 | -3.864 | 0.0021658 | 2.6644 |
| 18 | Indolelactic acid | 0.071133 | -3.8133 | 0.00029877 | 3.5247 |
| 19 | Inositol | 0.099563 | -3.3282 | 0.00024823 | 3.6051 |
| 20 | Valine | 0.11119 | -3.1689 | 0.0041015 | 2.3871 |
| 21 | Pyroglutamic acid | 0.11582 | -3.1101 | 0.0020834 | 2.6812 |
| 22 | Methane | 0.13316 | -2.9088 | 0.092594 | 1.0334 |
| 23 | Alanine | 0.1397 | -2.8395 | 0.00198 | 2.7033 |
| 24 | Isoleucine | 0.14106 | -2.8256 | 0.0038761 | 2.4116 |
| 25 | Norleucine | 0.16196 | -2.6263 | 0.022189 | 1.6539 |
| 26 | Myristic acid | 0.18663 | -2.4218 | 0.094608 | 1.0241 |
| 27 | GMP | 0.18969 | -2.3983 | 0.03882 | 1.4109 |
| 28 | Inositol 2-phosphate | 0.20178 | -2.3092 | 0.00095619 | 3.0195 |
| 29 | Hydroxyphenyllactic acid | 0.23199 | -2.1079 | 0.087787 | 1.0566 |
| 30 | Ornithine | 0.26935 | -1.8924 | 0.023762 | 1.6241 |
| 31 | Pentadecanoic acid | 0.27869 | -1.8433 | 0.00087115 | 3.0599 |
| 32 | Serotonin | 0.33016 | -1.5988 | 0.017615 | 1.7541 |
| 33 | 2-Hydroxybutyric acid | 0.34146 | -1.5502 | 0.026569 | 1.5756 |
| 34 | Hypoxanthine | 0.35364 | -1.4996 | 0.033843 | 1.4705 |
| 35 | Glucose-1-phosphate | 0.35477 | -1.495 | 0.053084 | 1.275 |
| 36 | Linoleic Acid | 0.36335 | -1.4606 | 0.010452 | 1.9808 |
| 37 | Thymine | 0.37602 | -1.4111 | 0.049052 | 1.3093 |
| 38 | Eicosenoic acid | 0.38316 | -1.384 | 0.012358 | 1.9081 |
| 39 | Oleic acid | 0.39624 | -1.3355 | 0.019878 | 1.7016 |
| 40 | Cholesterol | 0.4554 | -1.1348 | 0.034247 | 1.4654 |
| 41 | 3-Sulfinoalanine | 0.5302 | -0.9154 | 0.07285 | 1.1376 |
| 42 | 5-Hydroxy-L-tryptophan | 1.821 | 0.86475 | 0.021777 | 1.662 |
| 43 | Cinnamic acid | 1.8773 | 0.90866 | 0.087286 | 1.0591 |
| 44 | Mandelic acid | 2.2668 | 1.1806 | 0.054271 | 1.2654 |
| 45 | Docosahexaenoic acid | 4.1045 | 2.0372 | 0.096563 | 1.0152 |
| 46 | Phytosphingosine | 5.9564 | 2.5745 | 0.072494 | 1.1397 |
| 47 | ƴ-Linolenic acid | 18.467 | 4.2069 | 0.00067789 | 3.1688 |
| 48 | Galactose | 43.054 | 5.4281 | 0.0012646 | 2.898 |

## Targeted Lipidomics

**Table S 11:** List of **lipids** identified by targeted LC-MS/MS

| **No** | **Lipids** | **Classes/Subclasses** | |
| --- | --- | --- | --- |
|  |  | **Sterols** | |
| 1 | CE 15:0 | Chol. Ester | Cholesterol ester |
| 2 | Cholesterol | Cholesterol | Cholesterol |
|  |  | **Glycerolipids** | |
| 3 | MG (18:1) | MAG | Monoacylglycerol |
| 4 | DG (15:0_18:1) | DAG | Diacylglycerol |
| 5 | DG (18:3_20:5) | DAG | Diacylglycerol |
| 6 | DG (22:1_22:1) | DAG | Diacylglycerol |
| 7 | TG (15:0-18:1-15:1) | TAG | Triacylglycerol |
| 8 | TG (18:1/12:0/18:1) | TAG | Triacylglycerol |
|  |  | **Sphingolipids** | |
|  |  | ***Ceramides*** | |
| 9 | GlcCer(d18:1/24:0) | HexCer | Hexosylceramide |
| 10 | LacCer(d18:1/12:0) | Hex2Cer | Dihexosylceramide |
| 11 | LacCer(d18:1/22:0) | Hex2Cer | Dihexosylceramide |
| 12 | SM(d16:1/16:0) | SM | Sphingomyelin |
| 13 | SM(d18:0/16:1) | SM | Sphingomyelin |
| 14 | SM(d18:0/22:1) | SM | Sphingomyelin |
| 15 | SM(d18:0/24:0) | SM | Sphingomyelin |
| 16 | SM(d18:0/24:1) | SM | Sphingomyelin |
|  |  | **Glycerophospholipids** | |
|  |  | ***Phosphatidylcholines*** | |
| 17 | PC(14:0/14:1) | PC | Phosphatidylcholine |
| 18 | PC(14:0/16:0) | PC | Phosphatidylcholine |
| 19 | PC(14:0/22:5) | PC | Phosphatidylcholine |
| 20 | PC(14:1/18:1) | PC | Phosphatidylcholine |
| 21 | PC(15:0/20:5) | PC | Phosphatidylcholine |
| 22 | PC(18:0/14:1) | PC | Phosphatidylcholine |
| 23 | PC(18:0/20:5) | PC | Phosphatidylcholine |
| 24 | PC(18:1/18:1) | PC | Phosphatidylcholine |
| 25 | PC(18:1/20:5) | PC | Phosphatidylcholine |
| 26 | PC(18:2/18:1) | PC | Phosphatidylcholine |
| 27 | PC(18:2/22:6) | PC | Phosphatidylcholine |
| 28 | PC(18:3/20:5) | PC | Phosphatidylcholine |
| 29 | PC(24:0/P-18:1) | PC | Phosphatidylcholine |
| 30 | PC (P-16:0/14:1) | P-PC | Phosphatidylcholine plasmalogen |
| 31 | PC(24:0/P-18:1(11Z)) | P-PC | Phosphatidylcholine plasmalogen |
| 32 | PC(18:1(11Z)/P-16:0) | P-PC | Phosphatidylcholine plasmalogen |
| 33 | LPC (14:1) | LPC | Lysophosphatidylcholine |
| 34 | LPC (16:0) | LPC | Lysophosphatidylcholine |
| 35 | LPC (16:1) | LPC | Lysophosphatidylcholine |
| 36 | LPC (18:1) | LPC | Lysophosphatidylcholine |
| 37 | LPC (20:3) | LPC | Lysophosphatidylcholine |
| 38 | LPC (20:4) | LPC | Lysophosphatidylcholine |
| 39 | LPC (26:0) | LPC | Lysophosphatidylcholine |
| 40 | LPC (P-18:0) | P-LPC | Lysophosphatidylcholine plasmalogen |
| 41 | PC(O-16:0/16:1) | PC-O | Ether-linked Phosphatidylcholine |
| 42 | PC(O-16:0/18:2) | PC-O | Ether-linked Phosphatidylcholine |
| 43 | PC(O-18:0/20:4) | PC-O | Ether-linked Phosphatidylcholine |
| 44 | PC(O-18:1/18:1) | PC-O | Ether-linked Phosphatidylcholine |
| 45 | PC(O-18:1/18:2) | PC-O | Ether-linked Phosphatidylcholine |
| 46 | PC(O-18:1/20:0) | PC-O | Ether-linked Phosphatidylcholine |
| 47 | PC(O-18:2/20:0) | PC-O | Ether-linked Phosphatidylcholine |
| 48 | PC(O-20:0/18:3) | PC-O | Ether-linked Phosphatidylcholine |
| 49 | PC(O-20:0/20:4) | PC-O | Ether-linked Phosphatidylcholine |
| 50 | PC(O-20:1/20:4) | PC-O | Ether-linked Phosphatidylcholine |
|  |  | ***Phosphatidylethanolamine*** | |
| 51 | PE (14:0/18:2) | PE | Phosphatidylethanolamine |
| 52 | PE (15:0_18:1) | PE | Phosphatidylethanolamine |
| 53 | PE (16:1/22:6) | PE | Phosphatidylethanolamine |
| 54 | PE (18:3/16:0) | PE | Phosphatidylethanolamine |
| 55 | PE (P-16:0/20:5) | P-PE | Phosphatidylethanolamine plasmalogen |
| 56 | LPE (18:0) | LPE | Lysophosphatidylethanolamine |
| 57 | LPE (18:1) | LPE | Lysophosphatidylethanolamine |
| 58 | LPE (20:4) | LPE | Lysophosphatidylethanolamine |
| 59 | LPE (22:4) | LPE | Lysophosphatidylethanolamine |
| 60 | LPE (22:6) | LPE | Lysophosphatidylethanolamine |
|  |  | ***Phosphatidylglycerol*** | |
| 61 | PG (15:0_18:1) | PG | Phosphatidylglycerol |
| 62 | PG (18:0/18:1) | PG | Phosphatidylglycerol |
|  |  | ***Phosphatidylinositol*** | |
| 63 | PI (15:0_18:1) | PI | Phosphatidylinositol |
| 64 | PI (16:1/18:1) | PI | Phosphatidylinositol |
| 65 | PI (18:1/18:1) | PI | Phosphatidylinositol |
| 66 | PI (20:1/18:2) | PI | Phosphatidylinositol |
| 67 | PI (20:2/18:0) | PI | Phosphatidylinositol |
| 68 | PI (20:4/18:0) | PI | Phosphatidylinositol |
| 69 | PIP(16:1(9Z)/16:1(9Z)) | PIP | Phosphatidylinositol phosphate |
| 70 | PIP(16:0/18:1(9Z)) | PIP | Phosphatidylinositol phosphate |
| 71 | PS (15:0/18:1) | PS | Phosphatidylserine |
| 72 | PS (16:1/20:0) | PS | Phosphatidylserine |

**Table S12:** Significantly altered lipids (n = 3) using MetaboAnalyst 6.0 (ANOVA & post-hoc Tests (FDR, q = 0.1; LSD, α = 0.05):

| **Lipids** | **f.**  **value** | **p.value** | **-LOG10(p)** | **FDR** | **Fisher's LSD** |
| --- | --- | --- | --- | --- | --- |
| LPC (14:1) | 372.5 | 6.27E-09 | 8.2 | 4.64E-07 | B[a]P - B[a]P+UV Control - B[a]P+UV UV - B[a]P+UV |
| PC (O-16:0/18:2) | 273.1 | 2.14E-08 | 7.7 | 7.93E-07 | B[a]P+UV - B[a]P B[a]P+UV - Control B[a]P+UV - UV |
| PC (15:0/20:5) | 215.8 | 5.44E-08 | 7.3 | 1.34E-06 | B[a]P+UV - B[a]P UV - B[a]P B[a]P+UV - Control B[a]P+UV - UV UV - Control |
| PC (14:0/22:5) | 127.9 | 4.25E-07 | 6.4 | 7.86E-06 | B[a]P+UV - B[a]P UV - B[a]P B[a]P+UV - Control B[a]P+UV - UV UV - Control |
| PC (18:1/18:1) | 87.4 | 1.87E-06 | 5.7 | 2.77E-05 | B[a]P - B[a]P+UV Control - B[a]P+UV UV - B[a]P+UV UV - Control |
| PC (18:0/20:5) | 76.2 | 3.18E-06 | 5.5 | 3.92E-05 | B[a]P+UV - B[a]P UV - B[a]P B[a]P+UV - Control B[a]P+UV - UV UV - Control |
| LPC (26:0) | 39.0 | 4.02E-05 | 4.4 | 0.0004 | B[a]P - B[a]P+UV Control - B[a]P+UV UV - B[a]P+UV |
| PC (O-20:1/20:4) | 31.9 | 8.49E-05 | 4.1 | 0.0008 | B[a]P - Control UV - B[a]P B[a]P+UV - Control UV - B[a]P+UV UV - Control |
| PI (16:1/18:1) | 29.9 | 0.00011 | 4.0 | 0.0008 | B[a]P+UV - B[a]P UV - B[a]P B[a]P+UV - Control B[a]P+UV - UV UV - Control |
| PC (14:0/16:0) | 29.8 | 0.00011 | 4.0 | 0.0008 | B[a]P - B[a]P+UV Control - B[a]P+UV UV - B[a]P+UV |
| PC (O-18:2/20:0) | 29.5 | 0.00011 | 3.9 | 0.0008 | B[a]P - B[a]P+UV Control - B[a]P+UV UV - B[a]P+UV |
| PC (O-18:0/20:4) | 24.3 | 0.00023 | 3.6 | 0.0014 | B[a]P+UV - B[a]P UV - B[a]P B[a]P+UV - Control UV - Control |
| PC (18:3/20:5) | 22.2 | 0.00031 | 3.5 | 0.0018 | B[a]P - B[a]P+UV Control - B[a]P+UV UV - B[a]P+UV |
| SM(d18:0/22:1) | 21.4 | 3.53E-04 | 3.5 | 0.0019 | B[a]P - B[a]P+UV Control - B[a]P+UV UV - B[a]P+UV |
| PIP(16:1(9Z)/16:1(9Z)) | 15.9 | 0.00099 | 3.0 | 0.0049 | B[a]P - B[a]P+UV Control - B[a]P+UV UV - B[a]P+UV |
| PC (O-16:1/14:1) | 14.6 | 0.00131 | 2.9 | 0.0061 | B[a]P - B[a]P+UV B[a]P - UV Control - B[a]P+UV UV - B[a]P+UV |
| PC (14:1/18:1) | 14.3 | 0.00142 | 2.8 | 0.0062 | B[a]P - B[a]P+UV B[a]P - UV Control - B[a]P+UV UV - B[a]P+UV |
| SM(d18:0/24:0 | 21.4 | 3.53E-04 | 3.5 | 0.0019 | B[a]P - B[a]P+UV Control - B[a]P+UV UV - B[a]P+UV |
| LPC (16:0) | 11.8 | 0.003 | 2.6 | 0.0101 | B[a]P+UV - B[a]P B[a]P - UV B[a]P+UV - Control B[a]P+UV - UV Control - UV |
| PC (18:0/14:1) | 11.6 | 0.003 | 2.6 | 0.0102 | B[a]P+UV - B[a]P B[a]P+UV - Control B[a]P+UV - UV |
| LPC (20:4) | 11.4 | 0.003 | 2.5 | 0.0104 | B[a]P+UV - B[a]P B[a]P+UV - Control B[a]P+UV - UV |
| PS (16:1/20:0) | 10.3 | 0.004 | 2.4 | 0.0137 | B[a]P+UV - B[a]P B[a]P+UV - Control B[a]P+UV - UV |
| PC (O-20:0/20:4) | 9.8 | 0.005 | 2.3 | 0.0149 | UV - B[a]P UV - B[a]P+UV UV - Control |
| PC (O-16:0/16:1) | 9.6 | 0.005 | 2.3 | 0.0156 | B[a]P+UV - B[a]P B[a]P - Control B[a]P+UV - Control B[a]P+UV - UV |
| PG (18:0/18:1) | 8.5 | 0.007 | 2.1 | 0.0208 | B[a]P+UV - B[a]P B[a]P+UV - Control B[a]P+UV - UV |
| PC (14:0/14:1) | 8.5 | 0.007 | 2.1 | 0.0208 | B[a]P - B[a]P+UV Control - B[a]P+UV UV - B[a]P+UV |
| PE (18:3/16:0) | 7.9 | 0.009 | 2.1 | 0.0243 | B[a]P+UV - B[a]P B[a]P+UV - Control B[a]P+UV - UV |
| SM(d16:1/16:0) | 12.1 | 0.002 | 2.6 | 0.0099 | B[a]P - B[a]P+UV Control - B[a]P+UV UV - B[a]P+UV |
| PC (18:2/18:1) | 6.8 | 0.014 | 1.9 | 0.0349 | B[a]P - B[a]P+UV Control - B[a]P+UV UV - B[a]P+UV |
| PC (O-20:0/18:3) | 6.6 | 0.015 | 1.8 | 0.0362 | B[a]P - B[a]P+UV Control - B[a]P+UV UV - B[a]P+UV |
| PIP(16:0/18:1(9Z)) | 6.5 | 0.015 | 1.8 | 0.0362 | B[a]P - B[a]P+UV Control - B[a]P+UV UV - B[a]P+UV |
| PC (18:1/20:5) | 5.9 | 0.020 | 1.7 | 0.0455 | B[a]P+UV - B[a]P B[a]P+UV - Control UV - Control |
| PC (18:2/22:6) | 5.7 | 0.022 | 1.7 | 0.0482 | B[a]P+UV - B[a]P B[a]P+UV - Control B[a]P+UV - UV |

**Table S13:** Significantly altered **lipids** (FC < 0.666 or FC > 1.5) identified in U937 cells exposed to **UV** irradiation (n = 3) using volcano plot analysis in MetaboAnalyst 6.0.

| **No.** | **Lipids** | **FC** | **log2(FC)** | **raw.pval** | **-log10(p)** |
| --- | --- | --- | --- | --- | --- |
| 1 | PC (15:0/20:5) | 0.60225 | -0.73157 | 0.0010322 | 2.9862 |
| 2 | PC (O-18:0/20:4) | 0.63671 | -0.6513 | 0.0092452 | 2.0341 |

**Table S14:** Significantly altered **lipids** (FC < 0.666 or FC > 1.5) identified in U937 cells exposed to **4 µM B[a]P** (n = 3) using volcano plot analysis in MetaboAnalyst 6.0.

| **No.** | **Lipids** | **FC** | **log2(FC)** | **raw.pval** | **-log10(p)** |
| --- | --- | --- | --- | --- | --- |
| 1 | PE (18:3/16:0) | 0.52972 | -0.9167 | 0.022524 | 1.6473 |
| 2 | PE (P-16:0/20:5) | 0.49095 | -1.0263 | 0.045929 | 1.3379 |
| 3 | DG (18:3_20:5) | 3.0989 | 1.6317 | 0.088297 | 1.0541 |

**Table S15:** Significantly altered **lipids** (FC < 0.666 or FC > 1.5) identified in U937 cells exposed to **4 µM B[a]P and UV** irradiation (n = 3) using volcano plot analysis in MetaboAnalyst 6.0.

| **No.** | **Lipids** | **FC** | **log2(FC)** | **raw.pval** | **-log10(p)** |
| --- | --- | --- | --- | --- | --- |
| 1 | PC (15:0/20:5) | 0.23568 | -2.0851 | 1.706E-05 | 4.7681 |
| 2 | LPC (14:1) | 11.932 | 3.5768 | 2.398E-05 | 4.6201 |
| 3 | PC (O-16:0/18:2) | 0.5197 | -0.94424 | 3.084E-05 | 4.511 |
| 4 | SM (18:0;O2/22:1) | 3.4111 | 1.7703 | 1.330E-04 | 3.8761 |
| 5 | PC (14:0/22:5) | 0.53596 | -0.89981 | 1.361E-04 | 3.8661 |
| 6 | PC (18:0/20:5) | 0.37473 | -1.4161 | 2.691E-04 | 3.57 |
| 7 | SM (d18:0/24:0) | 3.1888 | 1.673 | 6.181E-04 | 3.2089 |
| 8 | PC (O-18:0/20:4) | 0.52879 | -0.91923 | 1.320E-03 | 2.8795 |
| 9 | PI (16:1/18:1) | 0.57809 | -0.79064 | 3.228E-03 | 2.491 |
| 10 | LPC (26:0) | 2.535 | 1.342 | 3.293E-03 | 2.4824 |
| 11 | PC (18:3/20:5) | 1.8491 | 0.88678 | 3.950E-03 | 2.4034 |
| 12 | LPC (20:4) | 0.34156 | -1.5498 | 4.261E-03 | 2.3705 |
| 13 | PC (14:0/14:1) | 1.9917 | 0.99402 | 4.840E-03 | 2.3152 |
| 14 | PC (14:0/16:0) | 3.4307 | 1.7785 | 5.134E-03 | 2.2895 |
| 15 | PC (O-18:2/20:0) | 1.8218 | 0.86534 | 5.683E-03 | 2.2454 |
| 16 | PC (O-16:1/14:1) | 2.0437 | 1.0312 | 9.394E-03 | 2.0272 |
| 17 | SM( d16:1/16:0) | 1.9555 | 0.96751 | 9.397E-03 | 2.027 |
| 18 | PIP(16:1(9Z)/16:1(9Z)) | 3.0828 | 1.6242 | 9.518E-03 | 2.0215 |
| 19 | PC (14:1/18:1) | 2.9067 | 1.5394 | 1.004E-02 | 1.9982 |
| 20 | LacCer (18:1;O2/12:0) | 2.8127 | 1.4919 | 2.087E-02 | 1.6805 |
| 21 | PE (18:3/16:0) | 0.41982 | -1.2522 | 2.693E-02 | 1.5698 |
| 22 | PC (18:2/22:6) | 0.57482 | -0.79883 | 2.731E-02 | 1.5637 |
| 23 | LPC( 20:3) | 0.34477 | -1.5363 | 2.732E-02 | 1.5635 |
| 24 | PS (16:1/20:0) | 0.509 | -0.97426 | 2.879E-02 | 1.5407 |
| 25 | PE (15:0_18:1) | 0.29137 | -1.7791 | 3.104E-02 | 1.5081 |
| 26 | PG (18:0/18:1) | 0.45145 | -1.1474 | 3.260E-02 | 1.4868 |
| 27 | PIP(16:0/18:1(9Z)) | 5.1211 | 2.3565 | 3.736E-02 | 1.4276 |
| 28 | LacCer (d18:1/22:0) | 0.10379 | -3.2682 | 4.698E-02 | 1.3281 |
| 29 | LPE (22:6) | 2.452 | 1.294 | 4.864E-02 | 1.313 |
| 30 | LPE (20:4) | 2.0264 | 1.0189 | 8.120E-02 | 1.0905 |
| 31 | DG (15:0_18:1) | 0.64669 | -0.62885 | 9.131E-02 | 1.0395 |
| 32 | Cholesterol | 0.4948 | -1.0151 | 9.577E-02 | 1.0188 |

## Toxicological Assays

**Table S17:** Cellular assay results showing average, standard deviation (SD), and standard error of the mean (SEM) for various treatments

|  | Average | SD | SEM | Assay |
| --- | --- | --- | --- | --- |
| Control | 100 | 7.99600821 | 1.78796179 | GSH/GSSG |
| B[a]P | 172.530768 | 81.5785717 | 18.2415232 |  |
| UV | 72.5234208 | 67.9901701 | 15.2030642 |  |
| B[a]P+UV | 637.821599 | 795.165871 | 177.804494 |  |
|  |  |  |  |  |
| Control | 100 | 8.50329505 | 1.90139458 | ROS |
| B[a]P | 131.204854 | 46.2101867 | 10.3329119 |  |
| UV | 78.6257974 | 17.1612039 | 3.83736185 |  |
| B[a]P+UV | 56.4553213 | 37.1782055 | 8.31329947 |  |
|  |  |  |  |  |
| Control | 100 | 14.4254642 | 2.94458555 | MDA |
| B[a]P | 89.1877863 | 28.6506791 | 5.84829537 |  |
| UV | 107.006364 | 11.1175278 | 2.26935587 |  |
| B[a]P+UV | 1287.53556 | 150.896316 | 30.8015815 |  |
|  |  |  |  |  |
| Control | 100 | 6.69235099 | 1.36607043 | NQO1 |
| B[a]P | 105.075459 | 26.1823146 | 5.3444426 |  |
| UV | 124.808981 | 22.7226198 | 4.63823534 |  |
| B[a]P+UV | 1190.18984 | 836.138967 | 170.676152 |  |
|  |  |  |  |  |
| Control | 100 | 5.84512256 | 1.19313065 | LDH |
| B[a]P | 95.7238867 | 4.01549592 | 0.81965967 |  |
| UV | 158.196185 | 39.1531801 | 7.99210942 |  |
| B[a]P+UV | 6439.82968 | 1321.70502 | 269.791907 |  |
|  |  |  |  |  |
| Control | 100 | 2.76551785 | 0.56450897 | MTT |
| B[a]P | 116.556536 | 14.3347631 | 2.92607127 |  |
| UV | 89.7382841 | 14.5643171 | 2.97292877 |  |
| B[a]P+UV | 8.16661578 | 4.27136921 | 0.87188959 |  |
|  |  |  |  |  |
| Control | 100 | 3.67691779 | 0.7505477 | MMP |
| B[a]P | 116.231887 | 17.6906608 | 3.61109102 |  |
| UV | 151.656649 | 29.9982169 | 6.12336039 |  |
| B[a]P+UV | 1682.30951 | 931.461965 | 190.133877 |  |
|  |  |  |  |  |
| Control | 6.92738086 | 2.20642649 | 1.27388093 | Comet |
| B[a]P | 19.259335 | 14.4852813 | 8.36308107 |  |
| UV | 2.81508448 | 1.37281359 | 0.9707258 |  |
| B[a]P+UV | 75.4752598 | 8.13973059 | 5.7556587 |  |

# **Supporting figures**

## Untargeted Metabolomics

### *Comparison of groups exposed to 4 µM B[a]P (high) and 4 µM B[a]P +UV versus control conditions*


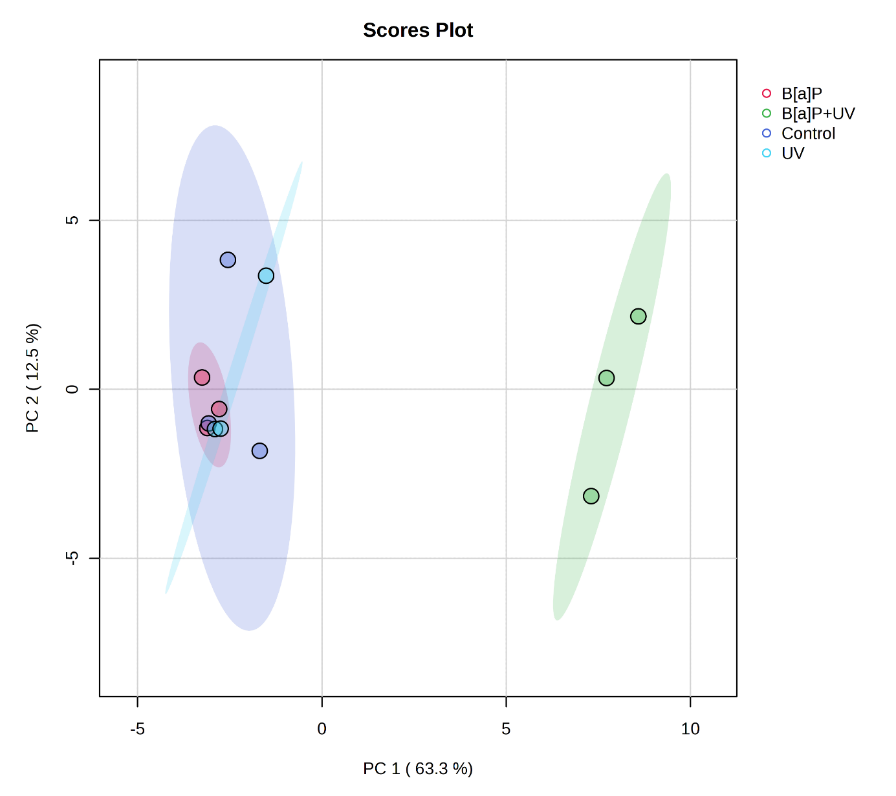


**Figure S1:** Principal component analysis (PCA) score plots of the first two principal components illustrating metabolite levels in the four groups: control condition, single exposure (UV and 4 µM B[a]P) and combined exposure (4 µM B[a]P+UV). PERMANOVA analysis confirms significant differences between the groups (F-value: 13.03, R-squared: 0.83, p-value: 0.017 based on 999 permutations).


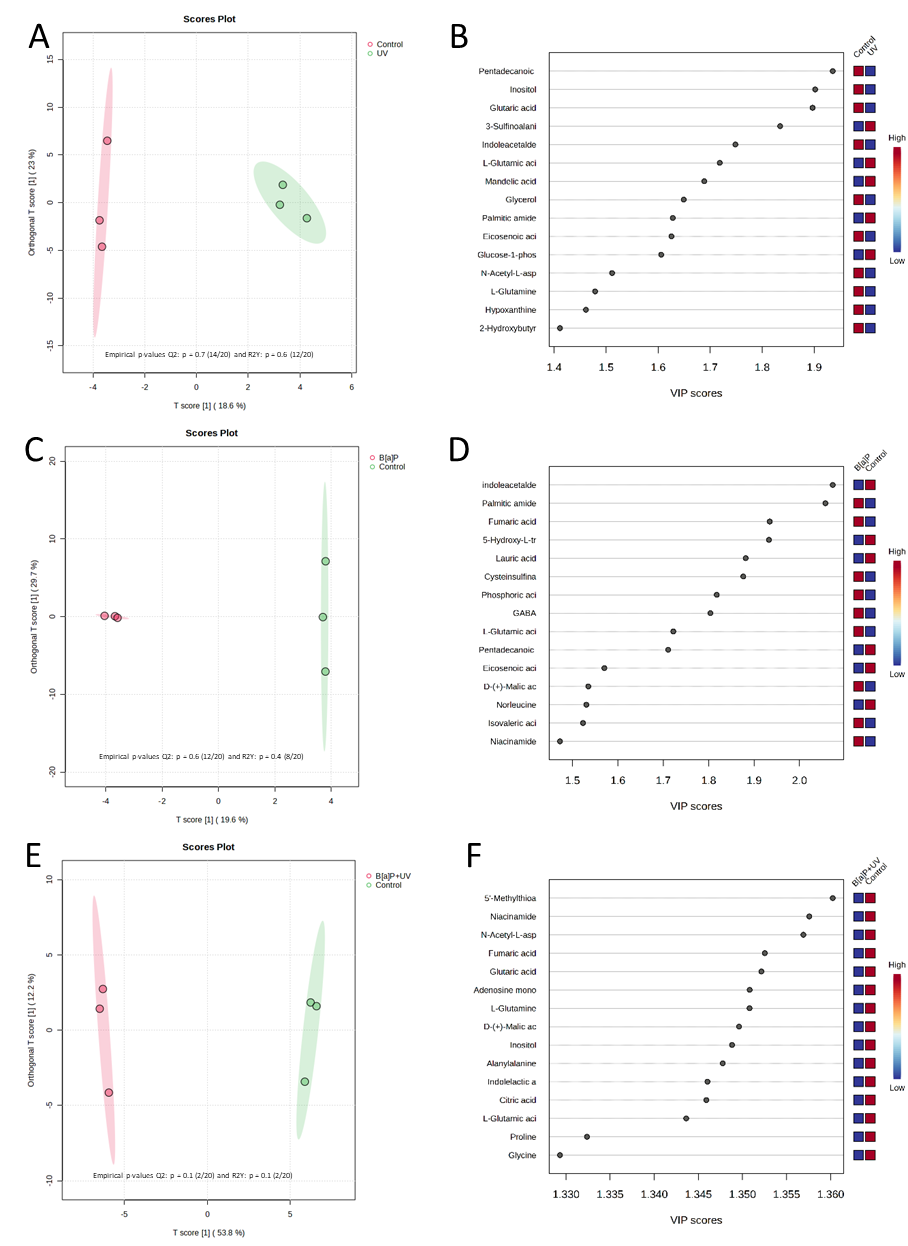


**Figure S2:** Orthogonal partial least squares discriminant analysis (OPLS-DA) score plots illustrate the metabolic differentiation between UV-irradiated samples (A), B[a]P-exposed samples (C), and samples exposed to both B[a]P and UV irradiation (E) compared to control conditions. The variable importance in projection (VIP) scores highlight the key metabolites driving these metabolic variations. A color-coded scale on the right side, ranging from blue to red, indicates the relative abundance of these metabolites, with blue representing low levels and red representing high levels (B, D, F).


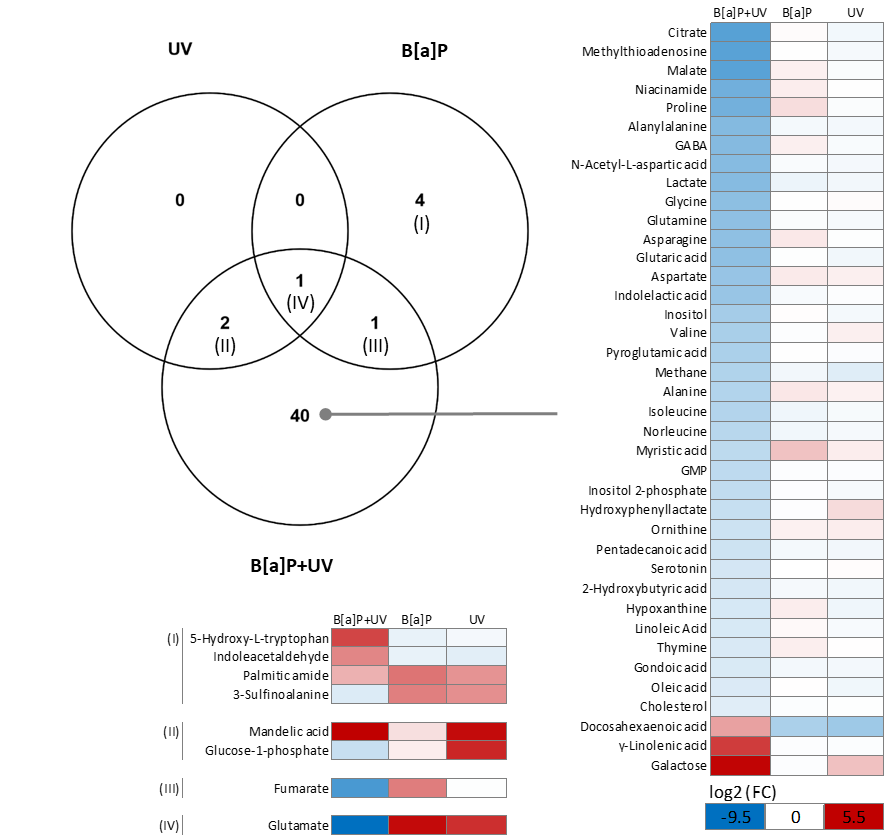


**Figure S3:** The Venn diagram illustrates the intersection and complement of regulated metabolites, as determined by volcano plots and OPLS-DA VIP scores. The heatmaps show the log2(FC) of the differentially expressed metabolites.


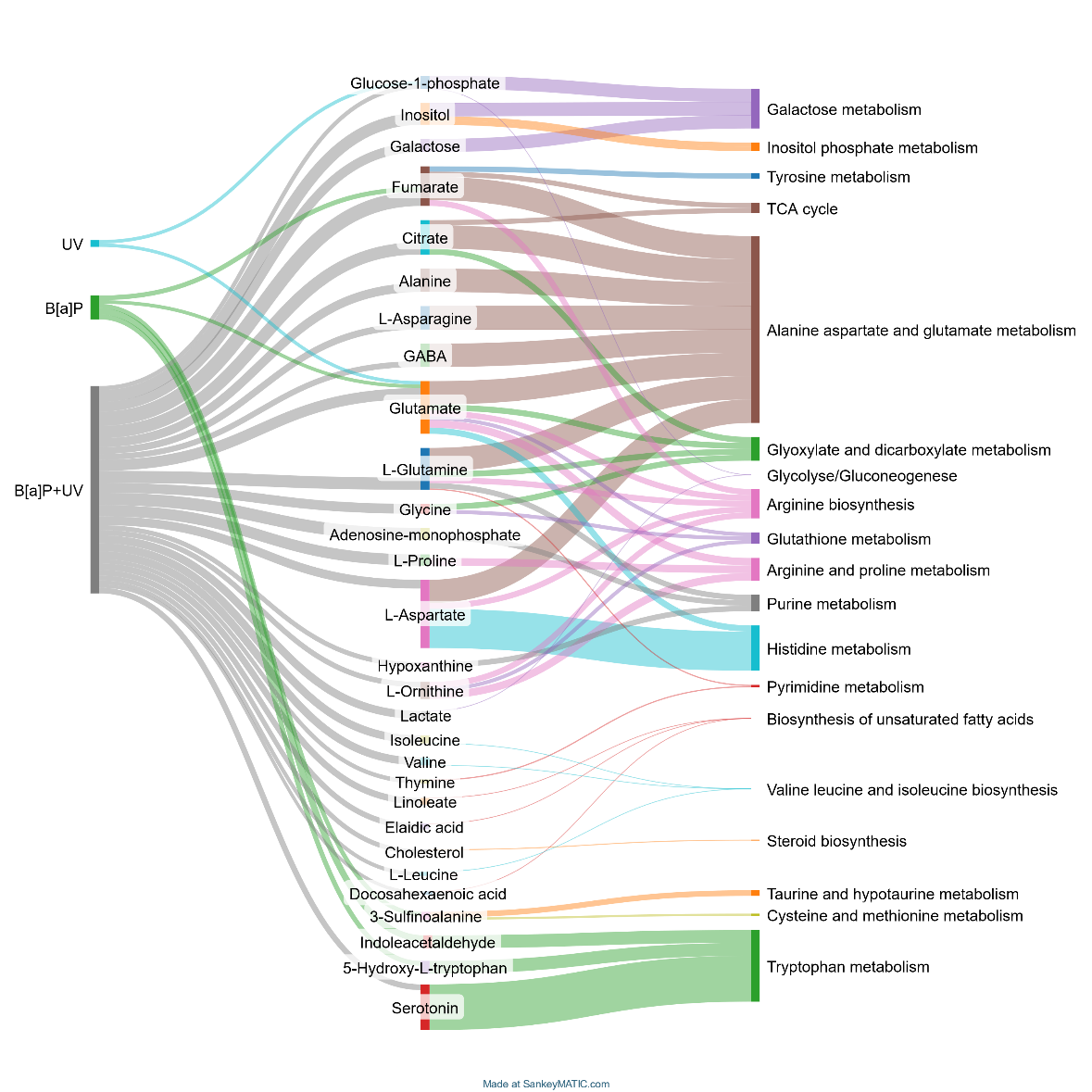


**Figure S4:** Sankey diagram visualizing the results of pathway enrichment analysis, highlighting the flow of significantly regulated metabolites (identified through volcano plot analysis) through significantly enriched metabolic pathways in the three exposure groups. The width of the flow represents the fold change of the significantly regulated metabolites and the impact of the metabolites on the pathways.

The Sankey diagram was based on the fold change values obtained from volcano plot analysis (Table S2-4) and pathway enrichment analysis (Table S5-7).


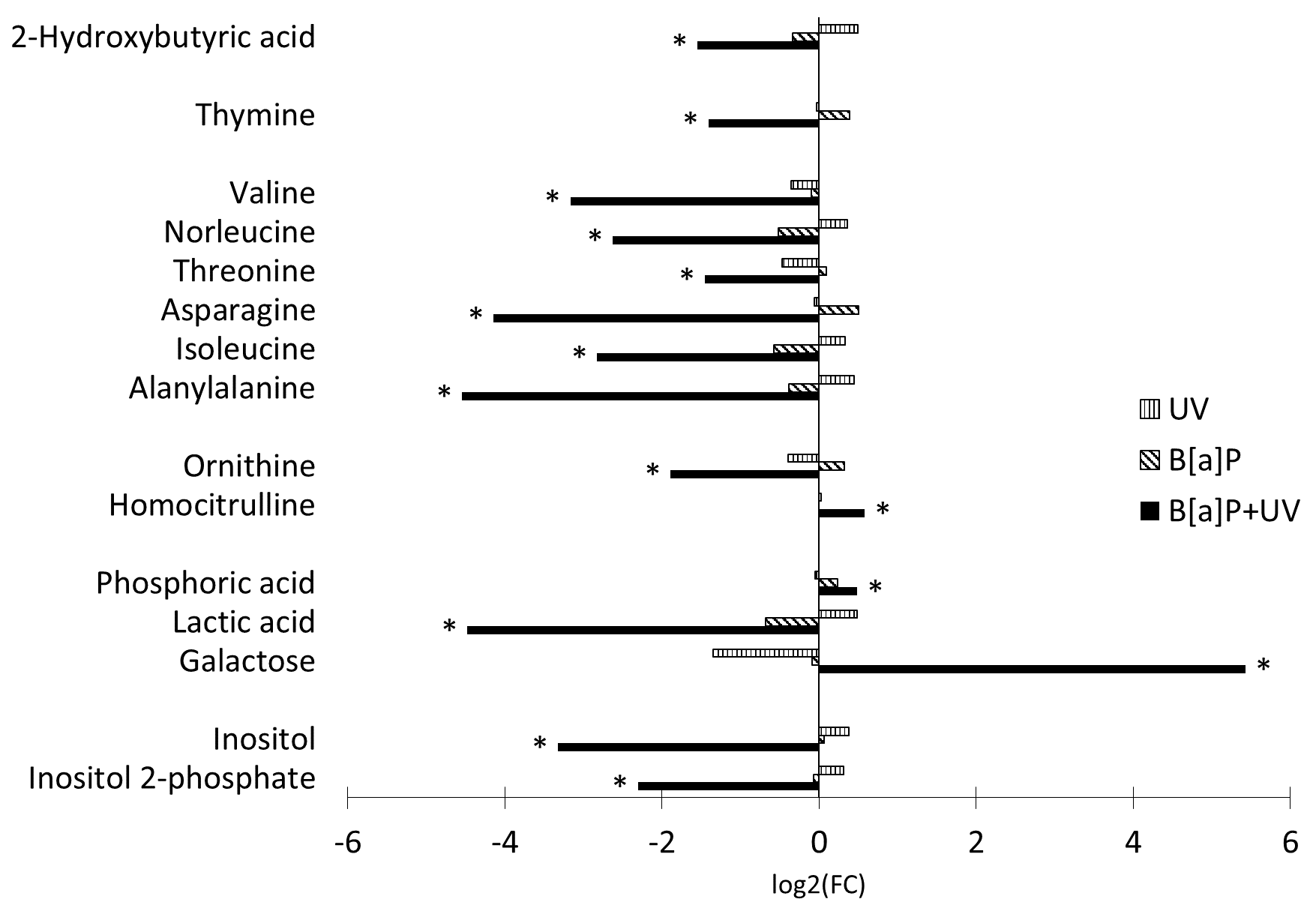


**Figure S5:** Bar charts of the log2 fold change of significantly altered metabolites (p < 0.05) not assigned to specific pathways ibetween the treatment and control. Statistical significance was determined using one-way ANOVA followed by post-hoc Fisher's LSD test.

### *Comparing the impact of combined B[a]P+UV exposure to control conditions across a range of B[a]P concentrations*


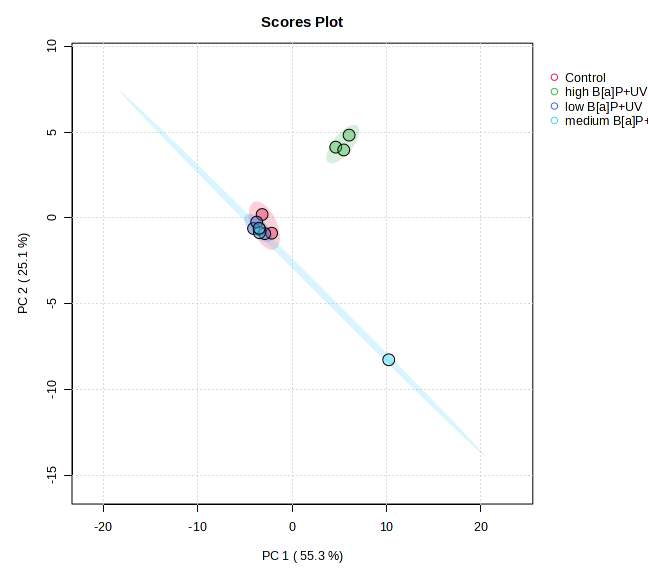


**Figure S6:** PCA score plots of the first two principal components illustrating metabolite levels following **B[a]P exposure** in the four groups: control condition, high (**4 µM**) B[a]P, low (**0.04 nM**) B[a]P and medium (**4 nM**) B[a]P combined with **UV** irradiation. PERMANOVA analysis confirms significant differences between the groups (F-value: 3.88, R-squared: 0.59, p-value: 0.01 based on 999 permutations).


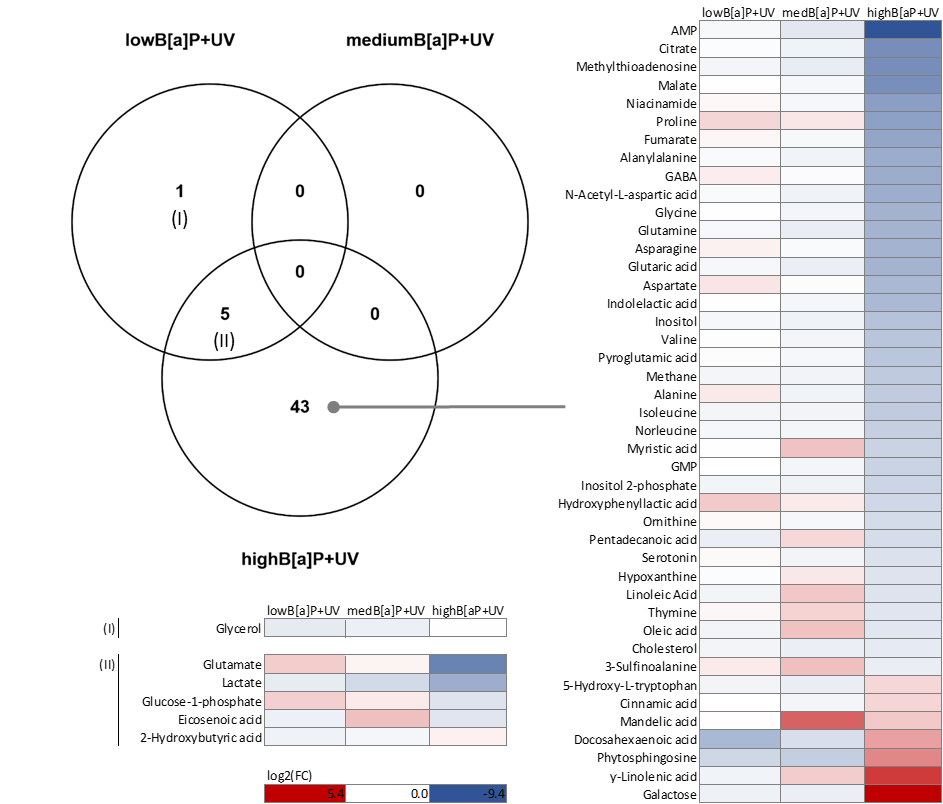


**Figure S7:** The Venn diagram illustrates the intersection and complement of regulated metabolites following B[a]P exposure in the three groups compared to control condition high (4 µM) B[a]P, low (0.04 nM) B[a]P and medium (4 nM) B[a]P combined with UV irradiation as determined by volcano plots. The heatmaps show the log2(FC) of the differentially expressed metabolites.

## Targeted Lipidomics


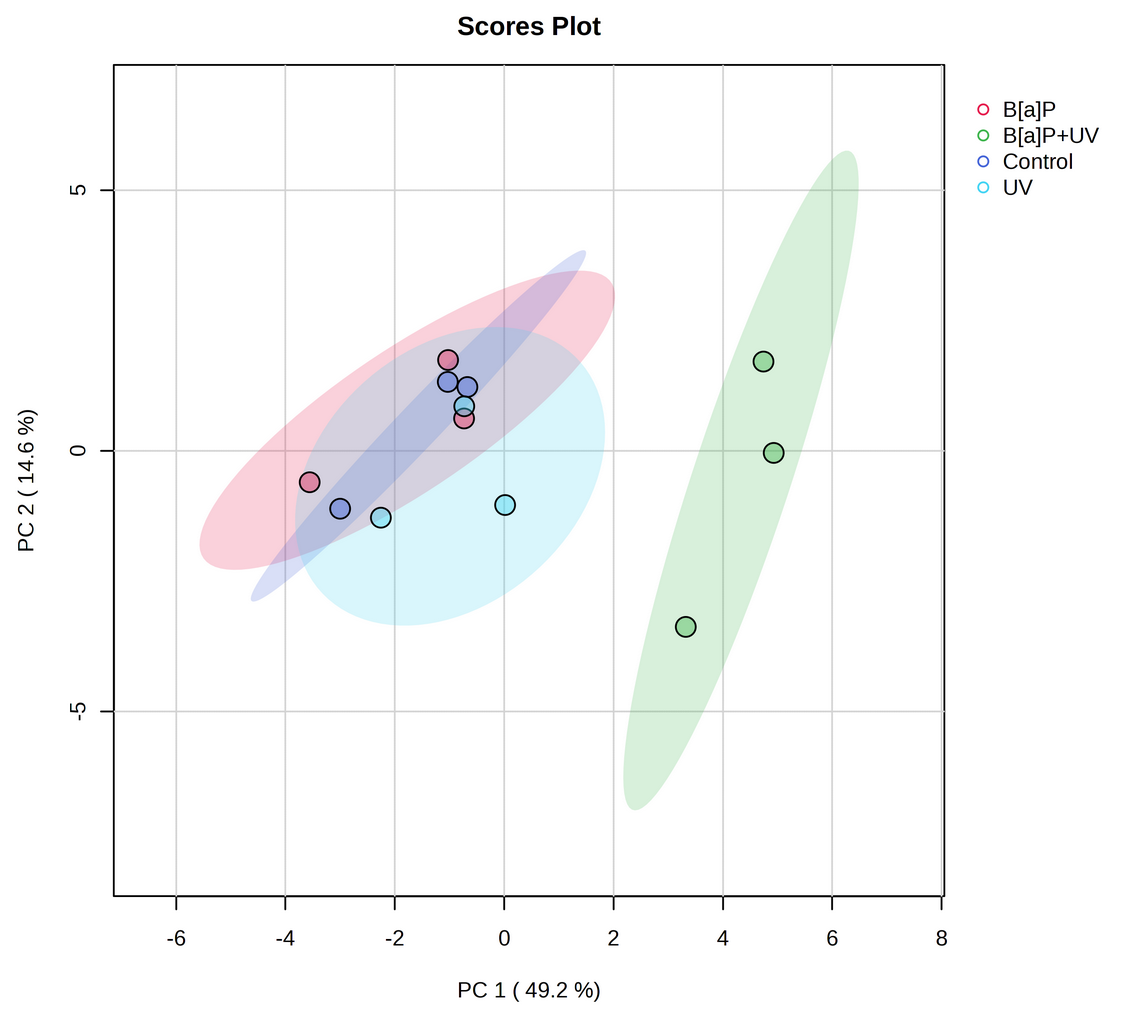


**Figure S8:** PCA score plots of the first two principal components illustrating **lipid** levels in the four groups: control condition, single exposure (UV and B[a]P) and combined exposure (B[a]P+UV). PERMANOVA analysis confirms significant differences between the groups (F-value: 6.076, R-squared: 0.69498, p-value: 0.01 based on 999 permutations).


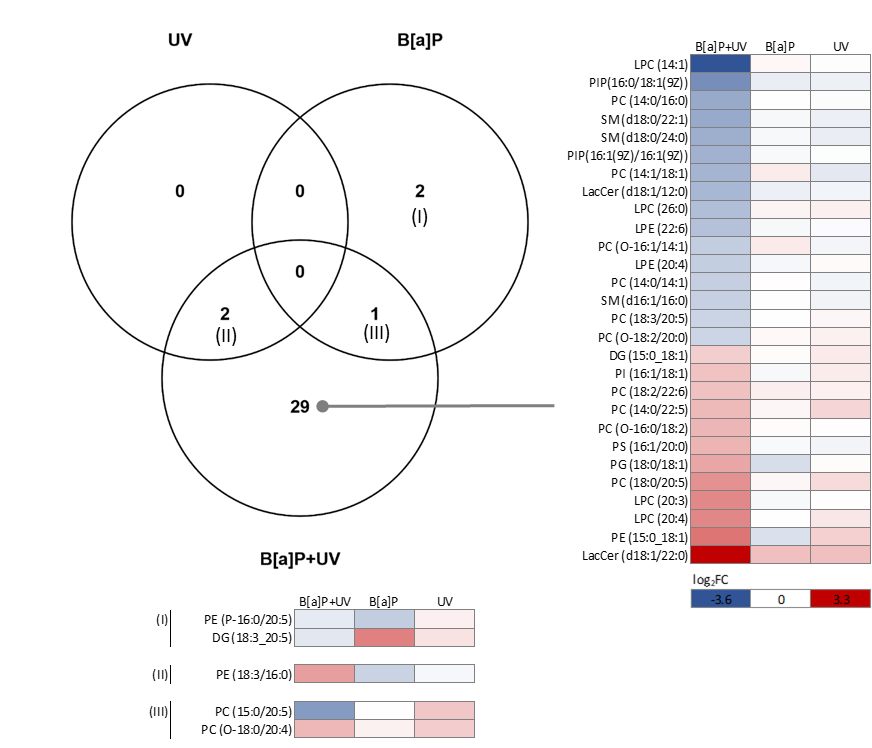


**Figure S9:** The Venn diagram illustrates the intersection and complement of regulated lipids, as determined by volcano plots. The heatmaps show the log2(FC) of the differentially expressed lipids.


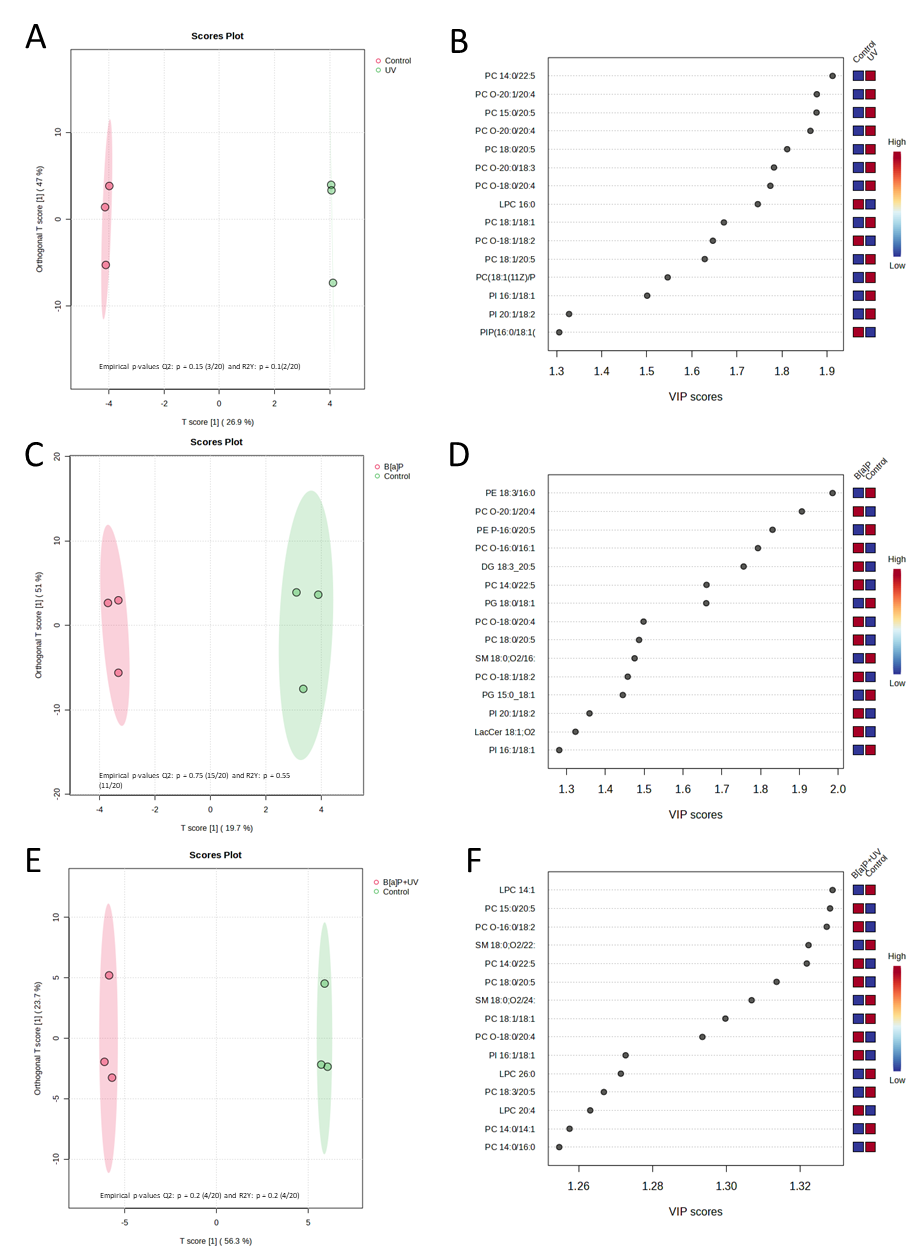


**Figure S10:** Orthogonal partial least squares discriminant analysis (OPLS-DA) score plots illustrate the differentiation in **lipids** between UV-irradiated samples (A), B[a]P-exposed samples (C), and samples exposed to both B[a]P and UV irradiation (E) compared to control conditions. The variable importance in projection (VIP) scores highlight the key lipids driving these variations. A color-coded scale on the right side, ranging from blue to red, indicates the relative abundance of these lipids, with blue representing low levels and red representing high levels (B, D, F).

**References:**

Cai S, Huo T, Li N, Xiong Z, Li F (2009) Lysophosphatidylcholine--biomarker of Metformin action: studied using UPLC/MS/MS. Biomed Chromatogr 23(7):782-6 doi:10.1002/bmc.1185

Hsu FF, Turk J (2001) Studies on phosphatidylglycerol with triple quadrupole tandem mass spectrometry with electrospray ionization: Fragmentation processes and structural characterization. Journal of the American Society for Mass Spectrometry 12(9):1036-1043 doi:Doi 10.1016/S1044-0305(01)00285-9

Kersch C, Masutin V, Alsaleh R, Schmitz-Spanke S (2025) Benzo[a]pyrene and UV light co-exposure: differential effects on oxidative stress and genotoxicity in human keratinocytes and ex vivo skin. Archives of toxicology doi:10.1007/s00204-025-04098-w

Masutin V, Kersch C, Schmitz-Spanke S (2022) A systematic review: metabolomics-based identification of altered metabolites and pathways in the skin caused by internal and external factors. Exp Dermatol 31(5):700-714 doi:10.1111/exd.14529

Meinhardt M, Krebs R, Anders A, Heinrich U, Tronnier H (2008) Wavelength-dependent penetration depths of ultraviolet radiation in human skin. J Biomed Opt 13(4):044030 doi:10.1117/1.2957970

Milne S, Ivanova P, Forrester J, Alex Brown H (2006) Lipidomics: an analysis of cellular lipids by ESI-MS. Methods 39(2):92-103 doi:10.1016/j.ymeth.2006.05.014

Pi JJ, Wu X, Feng YF (2016) Fragmentation patterns of five types of phospholipids by ultra-high-performance liquid chromatography electrospray ionization quadrupole time-of-flight tandem mass spectrometry. Analytical Methods 8(6):1319-1332 doi:10.1039/c5ay00776c
